# Health assessment of snacks and desserts in Guizhou Province: Analysis of fatty acids and sugar content

**DOI:** 10.1371/journal.pone.0321857

**Published:** 2025-06-02

**Authors:** Lu Zou, Xianjie Fu, Junfei Huang, Wenzheng Liu, Liqiang Zhou, Yibing Zhou, Ye Lin, Liya Liu

**Affiliations:** Guizhou Provincial Center for Disease Control and Prevention, Guizhou, China; Lusofona University of Humanities and Technologies: Universidade Lusofona de Humanidades e Tecnologias, PORTUGAL

## Abstract

**Introduction:**

Dietary patterns, particularly the excessive consumption of snacks high in fats and sugars, remain a pivotal factor producing adverse impact on the global prevalence of obesity and chronic diseases. However, the current situation is that there is insufficient study on the nutritional value and health risks of local snacks and desserts of Guizhou Province. To fill this gap, this study aimed to analyze the fatty acid (FA) composition and sugar content of popular snacks and desserts in Guizhou Province.

**Methods:**

A comprehensive nutritional evaluation was conducted on local snacks and desserts in Guizhou province, with a focus on the FA profile and five sugars (glucose, fructose, sucrose, maltose, and lactose). The study examined how cooking methods (steaming, baking, frying) and food classification (traditional vs. non-traditional) influence the nutritional profiles of these foods, which are predominantly made from rice, wheat, and cream.

**Results:**

Rice-based foods, particularly those steamed using traditional methods, showed superior nutritional profiles. They had significantly lower crude fat (7.35±1.50 g/100g) and total FA (6.10±1.55 g/100g) compared to other methods. Trans fatty acid (TFA) content was minimal (0.0179±0.0137 g/100g), and atherogenic index (AI) and thrombogenic index (TI) were low at 0.19±0.07 and 0.38±0.27, respectively. Rice-based foods also had an acceptable sugar content with no lactose, suitable for lactose-intolerant individuals. Among the rice-based foods, Rice Tofu (RT) had the best performance with the lowest Crude Fat (5.98 g/100g), AI (0.04), TI (0.05), highest monounsaturated FA (MUFA) content (3.11 g/100g), polyunsaturated FA (PUFA) to saturated FA (SFA) ratio (8.48), and n-3PUFA/n-6PUFA ratio (0.25), along with acceptable sugar levels.

**Conclusion:**

The nutritional value of snacks varies widely due to differences in raw materials, cooking methods, and traditional preparation techniques. Traditional steamed rice products, especially RT, offer the best nutritional profile and can be promoted as representative healthy traditional foods in Guizhou Province.

## Introduction

Food safety and nutrition are two pillars of public health, yet experiencing ongoing challenges owing to pathogens, unhealthy dietary habits, the overconsumption of certain nutrients, etc. [1,2]. The current emphases of food safety are the prevention of contamination and maintenance of the absence of harmful substances. Notably, the nutritional properties of food play an equally critical role in determining its impact on human health [3,4]. There is an increasingly worrying rise in the global prevalence of obesity and chronic diseases in recent decades, exhibiting an intimate association with dietary patterns, particularly the excessive consumption of snacks high in fats and sugars [5,6]. This trend is especially pronounced in industrialized countries, where snacks have become a major source of calorie intake, posing a threat to the occurrence of obesity, cardiovascular diseases, type 2 diabetes, and cancer [7].

Sugars, particularly refined sugars, can enhance flavor and provide sensory satisfaction, which are hence a key component of many snacks [8]. Without doubt, sugars are essential for maintaining proper bodily functions, such as brain and muscle activity [9]. Their overconsumption can trigger serious health issues, including organ dysfunction and chronic diseases [10]. Additionally, lactose intolerance is defined as a common condition caused by lactase deficiency that may threat individuals consuming dairy-based snacks, often resulting in digestive discomfort and other adverse symptoms [11].

Fats, another major component of snacks, are essential for energy production, cell membrane structure, and the absorption of fat-soluble vitamins (A, D, E, and K). However, excessive intake of fats, particularly of saturated FA (SFA) and trans FA (TFA), may cause obesity, heart disease, stroke, and other health problems [12]. Dietary imbalance of different FAs can produce detrimental effects on health, while polyunsaturated FAs (PUFAs) can reduce cardiovascular risk and modulate immune function [13]. It highlights the importance of understanding the FA composition of snacks and its potential impact on public health.

In China, there have been significant changes in dietary habits, with an increasing reliance on processed foods and snacks, with the rapid economic development and urbanization [14]. Owing to this shift, great concerns have been raised about the nutritional quality of these foods and their contribution to the rising prevalence of diet-related chronic diseases [15]. Guizhou Province, located in Southwest China, is home to a diverse population with unique ethnic traditions and dietary practices. The traditional snacks and desserts in this region, shaped by its multi-ethnic culture and geographical environment, offer a rich tapestry of flavors and cultural significance. However, there is an absence of thorough assessment of the nutritional value and potential health risks of these local foods, particularly in ethnic minority regions.

The lack of scientific evaluation of nutritional components of snacks and desserts in Guizhou poses a significant challenge for public health. In order to identify potential health impacts and promote healthier dietary choices, there is a need to elucidate the sugar content and FA profiles of these foods. It is of particular significance in a region where traditional foods are deeply intertwined with cultural identity, and where the preservation of these culinary traditions must be balanced with the need to address modern health concerns.

To fill this gap described above, the present study was conducted to comprehensively assess the nutritional components of popular snacks and desserts in Guizhou, China. Focusing on the characterization of corresponding sugar content and FA profiles, the study will provide a scientific basis for understanding the relationship between the nutritional properties of snacks and their impact on health, and evaluating their specific health impacts. Eventually, it may facilitate consumers’ decision-making of dietary choices, and support the sustainable development of local food industries, thus promoting both public health and cultural preservation.

Findings of this study may have broader implications for food safety and nutrition policy both domestically and internationally. Firstly, it may benefit the formulation of more effective strategies by policymakers to prevent against diet-related chronic diseases. Furthermore, this research will contribute to the growing body of knowledge on the intersection of cultural heritage and modern nutrition science, offering valuable insights for promoting healthier dietary habits, while preserving traditional food practices.

## Materials and methods

### Materials

All chemicals used in this study (analytical grade) were purchased from Sinopharm Chemical Reagent Co. Ltd. (Shanghai, China), including hydrochloric acid, ammonium hydroxide, pyrogallic acid, petroleum ether (boiling range 30~60 ◦C), ethanol (95%), sodium hydroxide, boron trifluoride methanol solution (15%), anhydrous sodium sulfate, sodium chloride, sodium hydrogen sulfate, potassium hydroxide, absolute alcohol, ascorbic acid. The triglyceride standard, mixed fatty acid (FA) methyl ester standard (purity ≥ 97%), 38 kinds of single FA methyl ester standard, the standard of glucose, fructose, sucrose, maltose, and lactose were obtained from Solarbio (Beijing, China). Distilled water and pH indicator paper were prepared by As One (Shanghai, China)

### Samples and sample preparation

The top-46 popular snacks and desserts with the highest order volume in Guizhou Province according to data from Meituan, the largest food delivery platform in China in 2024. Three samples for each of this top-46 list, with three batches per sample, were obtained at supermarkets, local markets, restaurants, and fast-food chains. The samples were packed in Eppendorf tube for measurement after grinding using a laboratory homogenizer (MX16-waring-CB15K laboratory, Beyotime, Shanghai, China). Specific details are provided in Table 1.

**Table 1:** The details of samples.

No. Item site	Dessert/Snack Name	Traditional/Common	Abbreviation	Processing method	Main component
**1**	Semi-cooked Cheesecake	Common	SCC	Baking	Cream
**2**	Taro Cream Cake	Common	TCC	Baking	Cream
**3**	Durian Mille-Feuille	Common	DMF	Baking	Cream
**4**	Rum and Grape Flavored Ice Cream Mooncake	Common	RGICM	Baking	Cream
**5**	Tiramisu	Common	TI	Steaming	Cream
**6**	Black Forest Cake	Common	BFC	Steaming	Cream
**7**	Xue Meiniang	Common	XMN	Steaming	Cream
**8**	Lime Cake	Common	LC	Steaming	Cream
**9**	Blueberry Cake	Common	BC	Steaming	Cream
**10**	Strawberry Flavored Ice Cream Mooncake	Common	SFICM	Steaming	Cream
**11**	Vanilla and Macadamia Nut Flavored Ice Cream Mooncake	Common	VMNFICM	Steaming	Cream
**12**	Mochi	Common	MO	Baking	Rice
**13**	Red Bean Bun	Common	RBB	Baking	Rice
**14**	Purple Rice Cake	Traditional	PRC	Baking	Rice
**15**	RICE CAKE	Traditional	RC	Baking	Rice
**16**	Crab Roe Crisps	Traditional	CRC	Frying	Rice
**17**	Sesame crisp	Traditional	SC	Frying	Rice
**18**	Taosu	Common	TS	Frying	Rice
**19**	Tangerine and Pomelo Flavored Coffee Mooncake	Common	TPFM	Steaming	Rice
**20**	Cocoa Flavored Coffee Mooncake	Common	CFCM	Steaming	Rice
**21**	Coconut and Apple Mooncake with Litsea Cubeba Flavor	Traditional	CAMLC	Steaming	Rice
**22**	Golden Salad Creamy Yolk and Nut Mooncake	Common	GSCYNM	Steaming	Rice
**23**	Vanilla Flavored Coffee Mooncake	Common	VFCM	Steaming	Rice
**24**	Niu Dagun	Traditional	ND	Steaming	Rice
**25**	Rice Tofu	Traditional	RT	Steaming	Rice
**26**	Cotton Grass Rice Cake	Traditional	CGRC	Steaming	Rice
**27**	Zunyi Cake	Traditional	ZC	Baking	Wheat
**28**	Yuanzu Pineapple Cake	Traditional	YPC	Baking	Wheat
**29**	Cui Bobo Cake	Traditional	CBC	Baking	Wheat
**30**	Roasted Ham Mooncake	Traditional	RHM	Baking	Wheat
**31**	Hand-torn Bread	Common	HTB	Baking	Wheat
**32**	Dried Meat Floss Bread	Common	DMFB	Baking	Wheat
**33**	Puff Pastry	Common	PP	Baking	Wheat
**34**	Pineapple Bun	Common	PB	Baking	Wheat
**35**	Egg Roll	Common	ER	Baking	Wheat
**36**	Cookies	Common	CK	Baking	Wheat
**37**	Dried Meat Floss Bun	Common	DMFb	Baking	Wheat
**38**	Cake Roll	Common	CR	Baking	Wheat
**39**	Strawberry Magic Wand	Common	SMW	Baking	Wheat
**40**	Egg and Milk Toast	Common	EMT	Baking	Wheat
**41**	Muffin Cake	Common	MC	Baking	Wheat
**42**	Sandwich	Common	SD	Baking	Wheat
**43**	Coconut Cream	Common	CC	Baking	Wheat
**44**	Milk Flavored Wafers	Common	MFW	Baking	Wheat
**45**	Caramel Treats	Common	CT	Frying	Wheat
**46**	Donuts	Common	DN	Frying	Wheat

### Crude fat and FA profile

The determination of FA content was performed according to the first method, external standard method, of national standards GB 5009.168–2016 [16]. The crude fat extract was subjected to saponification and methylation. Then, the extract was analyzed using gas chromatography (GC) to quantitatively determine the content of FA methyl esters by the internal standard method. TFA, SFA, MUFA, and PUFA contents were calculated based on the FA content and conversion coefficients. The measurement was carried out in triplicate.

### The GC conditions

The carrier gas was high-purity nitrogen (N₂) at 2.0 mL/min. The injection port and flame ionization detector (FID) temperature were set at 250°C (split ratio: 20:1) and 300°C, respectively. The temperature program was: set at 120°C for 1 min initially, increase at 10°C/min to 175°C for 10 min, at 5°C/min to 210°C for 5 min, and finally at 5°C/min to 230°C for 25 min. The injection volume was 1.0 μL.

### Quality control

Determination of crude fat and FA required preliminary analysis of standard reference for milk powder. The sample analysis could proceed only after the results met the standard reference values. Each sample was determined in parallel twice, and then determined in parallel twice again under the same conditions at different times. This study took the mean value of the four independent determinations, and the absolute difference should not exceed 10% of the arithmetic mean.

### Sugars

Sugar content was determined according to national standards GB 5009.8–2023 with improvement [17].

### Preparation of solutions

The preparation initiated by weighing 5g of each sample into a 50 mL centrifuge tube. Centrifugation at 1,800 r/min for 15 min was performed after the addition, mixing, deflation and shaking of the 25 mL of petroleum ether. After that, petroleum ether was removed, with residual one evaporated. Then 30 mL of water was added and shaken for 5 min, after which 2 mL of zinc acetate solution and potassium ferrocyanide solution were slowly added, respectively. Next, the solutions were injected after 2 min of vortexing, 30 min of sonication and filtration through a PVDF.

### Instrument

Chromatographic separation was completed by a Angient LC system (1290 infinity II model), which included an autosampler, a degasser, a thermostatted column compartment, and a binary pump connected to a Angient 1290-infinity-II-ELSD model detector (USA). LabSolution 10.0(USA) was utilized for all system processing and data monitoring.

### Analytical procedure

During study design, fructose, glucose, sucrose, maltose, and lactose were planned to be determined using conventional isocratic elution. The mobile phase consisted of waterand acetonitrile at 70:30, with a flow rate of 1.00 mL min-1 and injection volume of 10 µL. Hypersil APS-2 column (5µm particle-size, 250 mm×4.6 mm id) was used for conducting separations, with the adjustment of column oven temperature at 40°C. The isocratic elution was continued for 13 min totally. The nitrogen pressure was 350 kPa, and the drift tube temperature 45°C.

### Quality control

Determination of sugars required preliminary analysis of standard reference for orange juice. Specific details were the same to those of determining FAs.

### Data analysis

Data, generated from three replicates, were averaged, sorted, and analyzed statistically using Excel 2016 and R language(version 9.4.191, R studio Corporation, Boston, BOS, USA). The mean values were calculated to ensure the reliability of the results. The variance heterogeneity was examined by Dunnett’s test. The total content of crude fat and FAs in Fujian specialty snacks was analyzed using correlation analysis and stratified non-parametric tests.

A sum of 38 sorts of FAs were measured, including 16 SFA, 9 MUFA, and 13 PUFA that included 8 n-6 PUFA, 5 n-3 PUFA. This study calculated the content and proportion of MUFA, TFA, PUFA/SFA ratio, n-3 PUFA/n-6 PUFA. The following two equations were employed to compute AI and TI [18]:


$$AI=\frac{C12:0+4\times C14:0+C16:0}{\sum MUFA-6PUFA+\sum N-3PUFA}$$ (1)

$$TI=\frac{C14:0+C16:0+C18:0}{0.5\times \sum MUFA+0.5\times n-3\times \sum n-3PUFA+\frac{\sum n-3PUFA}{\sum n-6PUFA}}$$ (2)

## Results

### Nutritional analysis of different sources of snacks and desserts

#### Crude fat and FA profiles.

**Fig 1:**
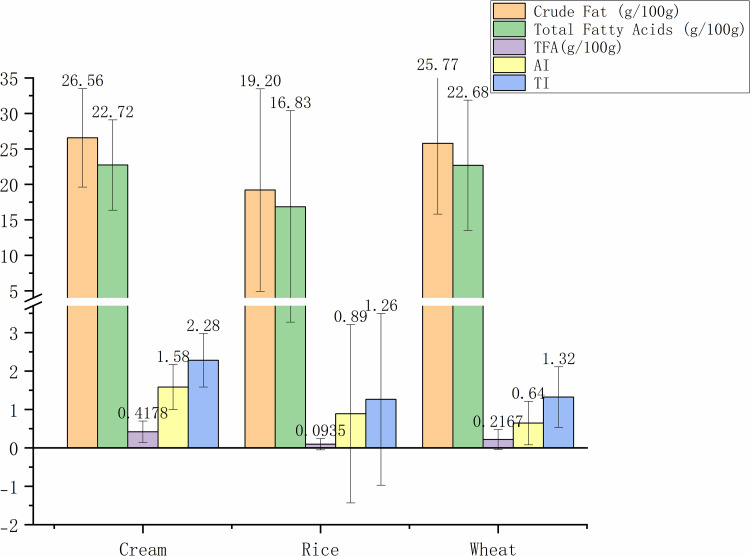
Crude fat, the total FA, TFA content, AI, TI of three kinds of snacks and desserts. FA, fatty acid; TFA, trans fatty acids; AI, atherogenic index; TI, thrombogenic index.

Fig 1 provides a detailed analysis of the FA composition in foods where cream, rice, and wheat serve as the primary ingredients. It illustrates the distribution of various FAs including crude fat, total FA,the TFA content, and the AI, TI value. Specifically, cream-based foods exhibited the highest levels of crude fat at 26.56±6.95 g/100g and total FA at 22.72±6.36 g/100g. Conversely, rice-based foods showed the lowest content of TFA at 0.0935±0.1459 g/100g, which is significant considering the health implications associated with high TFA intake. Wheat-based foods had a notable crude fat content of 25.77±9.97 g/100g and total FA at 22.68±9.18 g/100g, highlighting their substantial fat content as well. AI and TI values for cream-based foods were 1.58±0.59 and 2.28±0.69, respectively, indicating a higher potential risk for atherosclerosis and thrombosis.

**Fig 2:**
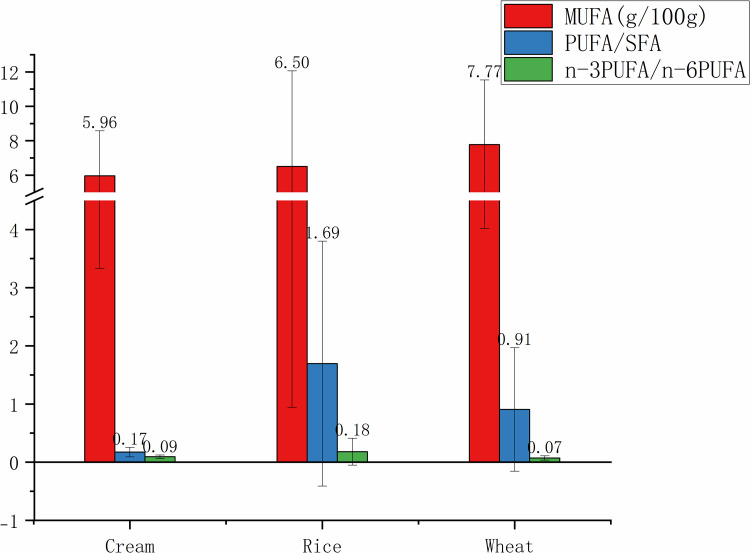
MUFA content, PUFA/SFA and n-3 PUFA/ n-6 PUFA of three kinds of snacks and desserts. MUFA, monounsaturated fatty acids; PUFA, polyunsaturated fatty acids; SFA, saturated fatty acids; PUFA/SFA, the ratio of PUFA to SFA; n-6 PUFA/ n-3 PUFA, the ratio of n-6 PUFA to n-3 PUFA.

In terms of PUFA (Fig 2), wheat-based foods had the highest MUFA content at 7.77±3.76 g/100g. Rice-based foods, however, stood out with the highest PUFA/SFA ratio at 1.69±2.11, indicating a more favorable balance of PUFA to SFA. It was an important nutritional clue as a higher PUFA/SFA ratio is generally associated with better cardiovascular health outcomes. Additionally, rice-based foods had an n-3PUFA/n-6PUFA ratio of 0.18±0.23, which, however, was not the highest among the three types of foods, still presenting a relatively balanced omega-3 to omega-6 FA ratio. AI and TI values for rice-based foods were 0.89±2.32 and 1.26±2.24, respectively, suggesting a lower risk of atherosclerosis and thrombosis compared to cream-based foods. Overall, rice-based foods may offer significant health benefits due to their lower TFA content, more favorable PUFA/SFA ratio, as well as lower AI and TI values, thus becoming a healthier option compared to cream- and wheat-based foods.

#### Sugar content.

**Fig 3:**
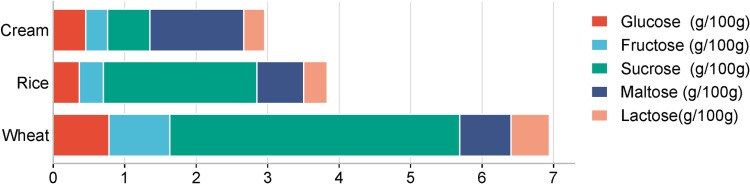
The content of five sugars of three kinds of snacks and desserts.

This study further conducted a comparative analysis of the sugar content in foods primarily composed of cream, rice, and wheat, with measurements in grams per 100 grams of food, as shown in the bar chart (Fig 3). Cream-based foods showed particularly high content in lactose, a predominant sugar in dairy products, while relatively low glucose and fructose levels. Rice-based foods were characterized by a high content of glucose, the most abundant sugar in this food type, along with moderate amounts of sucrose and maltose, and lower levels of fructose and lactose. Moreover, wheat-based foods exhibited a high content of sucrose, a significant source of this disaccharide, along with notable amounts of glucose and maltose, as well as lower quantities of fructose and lactose. Collectively, in view of the varied sugar profiles of these food types, cream-based foods may be a significant source of lactose, rice-based foods contribute substantially to glucose intake, and wheat-based foods provide a notable amount of sucrose. These data offer valuable insights for making informed dietary choices based on individual sugar requirements and preferences.

### Nutritional impact of different cooking methods on snacks and desserts

#### Crude fat and FA profile.

**Fig 4:**
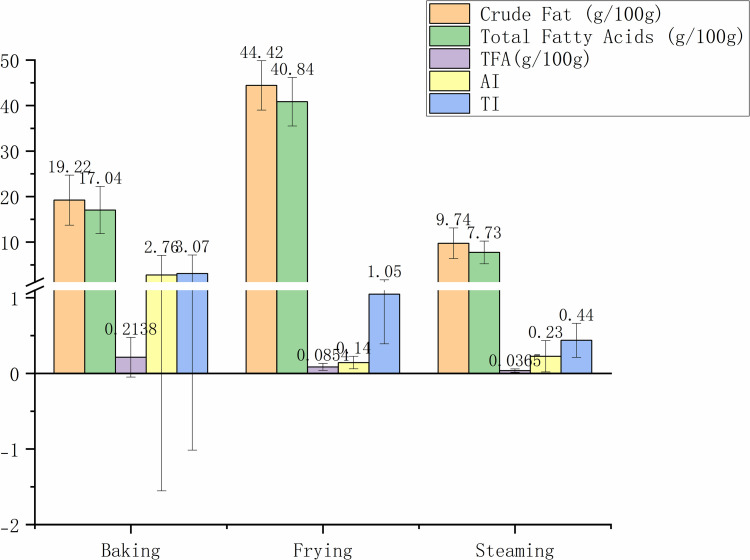
Crude fat, the total FA, TFA content, AI, TI of snacks and desserts through different cooking methods. FA, fatty acid; TFA, trans fatty acids; AI, atherogenic index; TI, thrombogenic index.

Fig 4 illustrates the impact of different cooking methods on the FA profile of rice-based foods. Baking yielded moderate levels of crude fat (19.22±5.49 g/100g) and total FA (17.04±5.18 g/100g), with relatively higher AI and TI index values (2.76±4.32 and 3.07±4.09, respectively). Frying significantly raised crude fat content to 44.42±5.43 g/100g and total FA to 40.84±5.32 g/100g, yet it minimized TFA and presented the lowest AI value. Steaming delivered the lowest fat content with 9.74±3.38 g/100g for crude fat and 7.73±2.50 g/100g for total FA, along with the lowest TFA content and TI values, suggesting that it may be the healthiest cooking method.

**Fig 5:**
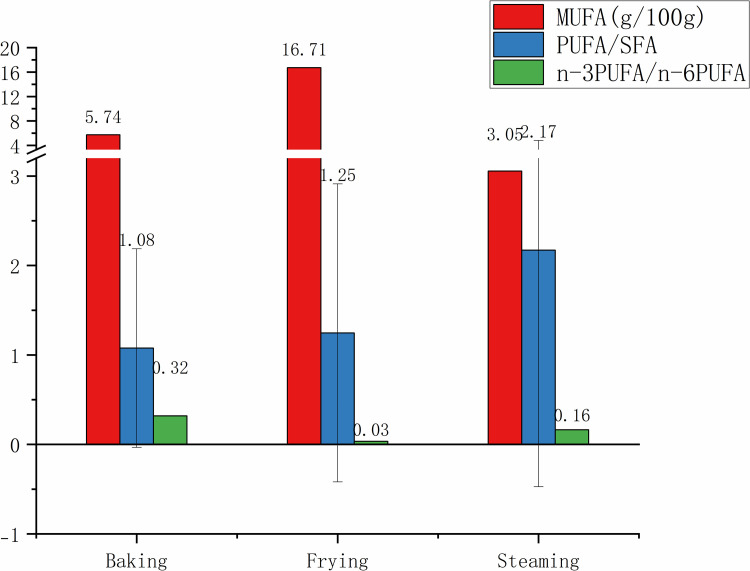
MUFA content, PUFA/SFA and n-3 PUFA/ n-6 PUFA of snacks and desserts through different cooking methods. MUFA, monounsaturated fatty acids; PUFA, polyunsaturated fatty acids; SFA, saturated fatty acids; PUFA/SFA, the ratio of PUFA to SFA; n-6 PUFA/ n-3 PUFA, the ratio of n-6 PUFA to n-3 PUFA.

Fig 5 examines the PUFA ratios across various cooking methods. Baking provided the lowest PUFA/SFA ratio (1.08±1.11) and the highest n-3PUFA/n-6PUFA ratio (0.32±0.44). Frying exhibited advantage in offering the highest MUFA content (16.71±1.54 g/100g), but less ideal yield of a very low n-3PUFA/n-6PUFA ratio (0.03±0.03). Steaming, despite its lower MUFA content (3.05±1.30 g/100g), produced the highest PUFA/SFA ratio (2.17±2.64) and a favorable n-3PUFA/n-6PUFA ratio (0.16±0.06). Collectively, while steaming can minimize fat content, it also maintains a beneficial balance of FAs, which can be an excellent cooking method for health-conscious consumers seeking to optimize their intake of healthy fats.

#### Sugar content.

**Fig 6:**
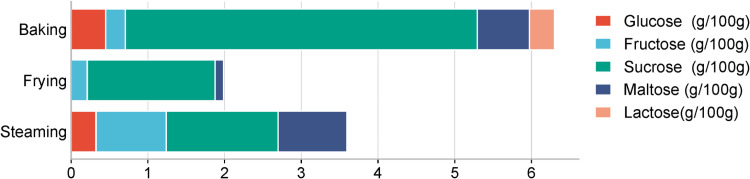
The content of five sugars of snacks and desserts through different cooking methods.

This study also constructed a bar chart (Fig 6) to present concise comparison of the sugar content across three cooking methods. Baking was associated with the highest levels of sucrose, likely due to added sweeteners, along with moderate amounts of glucose and maltose, and minimal lactose. Frying introduced the least sugar, with glucose being the most prevalent, followed by fructose, and almost no lactose. Steaming revealed a balanced sugar profile, with relatively high maltose and sucrose levels, and lower amounts of glucose and fructose. Therefore, the choice of cooking method has a profound impact on sugar intake, offering insights for consumers with demands for sugar consumption management.

### Nutritional impact of traditional vs. common cooking methods on snacks and desserts

#### Crude fat and FA profile.

**Fig 7:**
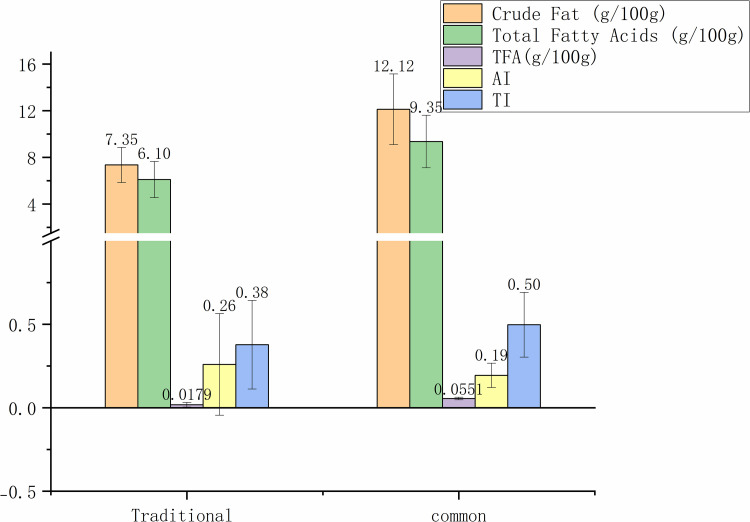
Crude fat, the total FA, TFA content, AI, TI of traditional and common snacks and desserts. FA, fatty acid; TFA, trans fatty acids; AI, atherogenic index; TI, thrombogenic index.

Fig 7 illustrates the FA composition in steamed rice-based foods prepared using traditional versus common methods. Traditional steaming resulted in lower crude fat (7.35±1.50 g/100g) and total FA(6.10±1.55 g/100g) compared to common methods (12.12±3.02 g/100g and 9.35±2.25 g/100g, respectively). Additionally, traditional method had lower TFA content (0.0179±0.0137 g/100g) and TI (0.38±0.27), indicating a potentially lower risk of cardiovascular diseases. In contrast, common methods showed slightly higher TFA (0.0551±0.0081 g/100g) and AI (0.19±0.07).

**Fig 8:**
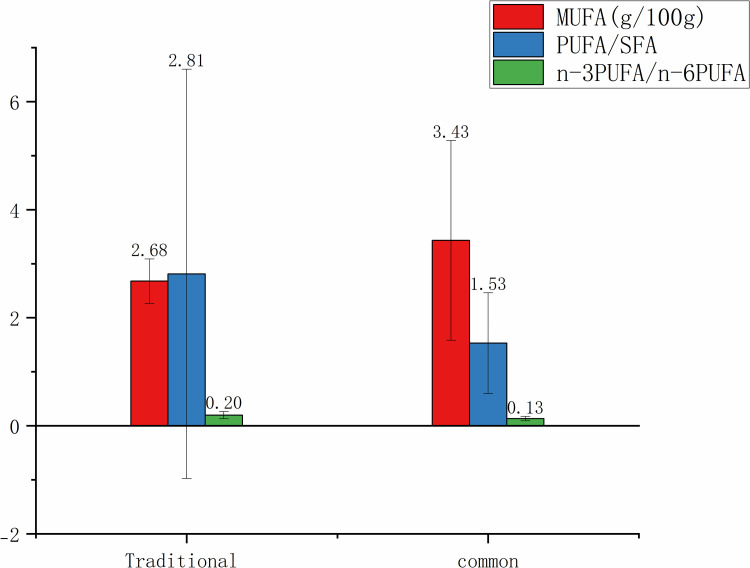
MUFA content, PUFA/SFA and n-3 PUFA/ n-6 PUFA. of traditional and common snacks and desserts. MUFA, monounsaturated fatty acids; PUFA, polyunsaturated fatty acids; SFA, saturated fatty acids; PUFA/SFA, the ratio of PUFA to SFA; n-6 PUFA/ n-3 PUFA, the ratio of n-6 PUFA to n-3 PUFA.

As presented in Fig 8 regarding PUFA ratios, traditional method provided a more favorable PUFA/SFA ratio (2.81±3.79) and a higher n-3PUFA/n-6PUFA ratio (0.20±0.07), suggesting a healthier balance of FAs. Common method, while having higher MUFA content (3.43±1.85 g/100g), exhibited a lower PUFA/SFA ratio (1.53±0.93) and a less favorable n-3PUFA/n-6PUFA ratio (0.13±0.04). Therefore, traditional steaming may be more beneficial for maintaining a healthy diet, particularly in terms of PUFA intake.

#### Sugar content.

**Fig 9:**
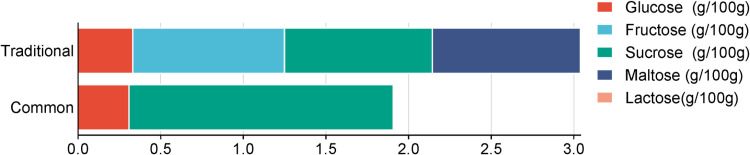
The content of five sugars of traditional and common snacks and desserts.

Sugar content between traditional and common food types, measured in grams per 100 grams, was also compared and is shown in Fig 9. Traditional foods had higher maltose content, suggesting a possible use of maltose-rich ingredients or fermentation in their preparation. By contrast, common foods were detected with higher sucrose levels, likely due to the frequent use of sugar as a sweetener. Both types of foods contained moderate amounts of glucose and fructose, with lactose being the least abundant in both, typically attributed to the reason that lactose is primarily found in dairy products. This analysis may facilitate our interpretation of different sugar profiles of traditional and common foods, aiding in making dietary choices based on sugar content preferences.

### Nutritional data of traditional rice-based foods through steaming method

Among these better food, which were Traditional rice-based foods through steaming method, RT had the best performance (Table 2 and 3). As depicted in Table 2, RT exhibits the lowest Crude Fat content at 5.98 g/100g and Total FA at 5.19 g/100g, which are undesirable indicators due to their association with increased cardiovascular risk. Additionally, although TFA was not detected in ND, RT had a small amount of TFA content at 0.0163 g/100g, which is another negative marker linked to heart disease. The AI and TI index for RT were also the lowest among the samples, at 0.04 and 0.05 respectively, suggesting a reduced propensity for atherosclerosis and thrombosis. Conversely, RT shows a high MUFA/SFA ratio at 8.48, which is a positive indicator. The PUFA/SFA ratio is also favorable in RT, indicating a balanced FA profile that may contribute to cardiovascular health. Furthermore, the n-3PUFA/n-6PUFA ratio in RT, presented a beneficial profile that supports anti-inflammatory effects and overall health.

**Table 2:** Crude fat content and FA Profile of four samples.

Name	Crude Fat (g/100g)	Total FA (g/100g)	TFA (g/100g)	AI	TI	MUFA (g/100g)	PUFA/SFA	n-3PUFA/n-6PUFA
**ND**	9.45	8.42	－	0.10	0.51	2.72	1.26	0.25
**CAMLC**	6.65	5.34	0.0328	0.71	0.29	2.12	0.82	0.16
**RT**	5.98	5.19	0.0163	0.04	0.05	3.11	8.48	0.25
**CGRC**	7.32	5.44	0.0223	0.18	0.66	2.77	0.69	0.12

FA, fatty acid; TFA, trans fatty acids; AI, atherogenic index; TI, thrombogenic index; MUFA, monounsaturated fatty acids; PUFA, polyunsaturated fatty acids; SFA, saturated fatty acids; PUFA/SFA, the ratio of PUFA to SFA; n-6 PUFA/ n-3 PUFA, the ratio of n-6 PUFA to n-3 PUFA. ND, Niu Dagun; CAMLC, Coconut and Apple Mooncake with Litsea Cubeba Flavor; RT, Rice Tofu; CGRC, Cotton Grass Rice Cake.

Note: indicates that the fatty acid was not detected, or that the content of fatty acid was below the detection limit of 0.0026 g/100g.

Table 3 illustrates that the sugar content across these samples is relatively low, and notably, lactose is absent in all samples, making them suitable for consumption by individuals with lactose intolerance. Although RT does not exhibit the lowest sugar content, its levels are still considerably low, which aligns with dietary preferences for reduced sugar intake.

**Table 3:** The content of five sugars of four samples.

Dessert/snacks Name	Glucose (g/100g)	Fructose (g/100g)	Sucrose (g/100g)	Maltose (g/100g)	Lactose (g/100g)	The total sugar (g/100g)
**ND**	0.648	－	－	0.291	－	0.939
**CAMLC**	0.160	－	0.895	1.501	－	2.557
**RT**	0.296	0.917	－	－	－	1.213
**CGRC**	0.225	－	－	－	－	0.225

ND, Niu Dagun; CAMLC, Coconut and Apple Mooncake with Litsea Cubeba Flavor; RT, Rice Tofu; CGRC, Cotton Grass Rice Cake.

Note: indicates that the sugar was not detected, or that the content of sugar was below the detection limit of 0.2g/100g

## Discussion

Dietary patterns, particularly the excessive consumption of snacks high in fats and sugars, remain a pivotal factor producing adverse impact on the global prevalence of obesity and chronic diseases [19,20]. However, the current situation is that there is insufficient study on the nutritional value and health risks of local snacks and desserts of Guizhou Province. The present study systematically analyzed the nutritional compositions of snacks and desserts made primarily from cream, rice, and wheat. Several common indicators, including total FA, TFA, MUFA, AI, TI, PUFA/SFA, n-3PUFA/n-6PUFA and sugar content, were incorporated to evaluate the nutritional value and health benefits of these foods.

Firstly, in terms of raw materials, rice-based foods showed better nutritional value. Rice contains rich carbohydrates, a small amount of fat, an appropriate amount of protein, and ideal FA composition. FAs in rice are mostly unsaturated FA, especially linoleic acid and linolenic acid, which are beneficial for maintaining cardiovascular health [21]. Consistently, this study found lower FA and TFA levels, as well as lower AI and TI in rice-based foods, supporting a lower risk of cardiovascular diseases. Similar to those reported in the existing research [21], our study highlights the advantages of rice products in terms of nutritional value and health benefits.

According to the recommendation of the World Health Organization (WHO), the total fat intake for adults should be less than 30% of total energy intake per day [22]. In this study, the average fat content of the three types of snacks and desserts was below this threshold. Consistently, a prior human study established a positive correlation between industrial TFA intake and the development of cardiovascular diseases [23]. Besides, the average TFA levels are below 0.35g/100g in snacks and desserts from different sources.

Moreover, MUFA works to lower cholesterol and low-density lipoprotein levels. Keeping a high PUFA/SFA ratio may contribute to the reduction of serum cholesterol and thus the incidence of atherosclerosis, while preventing heart disease. Moreover, PUFA/SFA>2 may exhibit a lipid-lowering effect, with more pronounced effect when the ratio increases [24]. The UK Department of Health recommends that the ratio of PUFA/SFA should be kept below 0.4 in human food intake [25]. In this study, cream- and rice-based foods had a ratio below 0.4 (0.17), and four times this threshold (1.69), respectively.

Based on the recommended FA intake for Chinese residents and their daily dietary patterns, the consumption of foods with n-3 PUFA is usually much lower than n-6 PUFA [26]. From a nutritional perspective, n-3 PUFA/n-6 PUFA is an available index for nutritional status assessment, with a higher ratio indicating higher nutritional and health value of the food. Simultaneously, n-3 PUFA/n-6 PUFA in daily diets should be controlled within the range of 0.1–0.2, according to the recommendations of the Food and Agriculture Organization of the United Nations and WHO [27]. In this study, the ratio of n-3 PUFA/n-6 PUFA was 0.15 in rice-based foods, aligning with international guidelines. In addition, rice-based foods in this study showed lower AI and TI values, suggesting potential greater benefits over other types of foods for cardiovascular health.

Furthermore, the method of processing is also a determinant of the nutritional composition of foods. Steaming, a traditional processing method, can maximize the retention of nutrients in foods, thus reducing nutrient loss. Significantly, the effective roles of steaming have been documented in retaining vitamins and minerals, while maintaining a good FA ratio in rice [28]. In this study, steamed rice products showed higher PUFA/SFA as well as lower AI and TI values. Therefore, steaming can effectively improve the FA composition of foods and reduce the risk of cardiovascular diseases. Additionally, steaming can reduce the sugar and calorie intake from foods, which may facilitate weight control and obesity-related disease prevention [29]. The data in this study also support this conclusion, with steamed rice products showing lower sugar content and better nutritional balance. Accordingly, rice products can maximize nutrient retention during steaming, reducing nutrient loss and possessing a more reasonable FA ratio [30].

Nutrient retention was also analyzed to further quantify the impact of traditional and modern cooking methods. Traditional steaming retained approximately 90% of vitamin B in rice, compared to 60–70% in baking, and preserved 85% of unsaturated FAs, versus 70% in frying [21]. These results support that traditional methods outperform modern cooking in the aspect of nutrient retention. Additionally, differences between traditional Chinese and Western foods have been highlighted in previous studies. For example, Chinese dim sum typically has lower calories and a more balanced nutritional profile than Western pastries, making it suitable for subjects pursuing low-fat and low-sugar diets [31]. Similarly, Western fermented sausages were reported to contain more flavor compounds than Chinese dried sausages, reflecting distinct processing methods [32]. Collectively, traditional Chinese foods, such as rice-based snacks studied here, offer unique nutritional and cultural benefits compared to Western processed foods, further supporting their value in promoting healthier dietary practices.

Simultaneously, cultural practices and preferences have significant impact on the consumption of snacks. Likewise, in Guizhou, rice-based snacks are an integral part of the dietary culture of ethnic minorities like the Miao and Dong people [33]. These snacks are enjoyed during festivals and ceremonies, holding deep cultural significance [34,35]. A similar pattern is also observed in South Asia, where food festivals play a crucial role in culture, with specific dishes served on particular occasions, which indicates cultural identity [36]. These traditions, to some extent, exhibit cultural connections and values, and food offerings in family gatherings bring pride and happiness. Despite their relatively low sugar and fat content, these snacks are favored for their health benefits as well as their symbolic value and traditional preparation methods, which are perceived as healthier and more natural [33]. This cultural context may offer an explanation for the enduring popularity of these snacks, even in the circumstance of the popularization of more modern, processed alternatives.

In addition, to provide a broader perspective, this study compared the nutritional profiles of the local snacks and desserts with similar products from other countries. Among the four rice-based foods prepared through traditional steaming, only ND (Niu Dagun) had slightly higher crude fat (9.45%) than the lowest Iranian sample, Sponge cake (8.99 ± 2.95%), while the other three samples had lower values [37]. Simultaneously, sugar content was also lower than most Iranian products, except for puffed products (0% sugar) [37]. Notably, all four rice-based foods exhibited lower TFA levels, potentially attributed stricter national standards in China. RT, in particular, demonstrated a total fat content comparable to Barakat’s snack (5.98g/100g vs. 4.97 g/100g), but with a PUFA/SFA ratio more than double (3.86) [38], highlighting its nutritional advantage. All together, traditional steaming and regional ingredients contribute to the superior quality of these foods. RT, a widely consumed snack in Guizhou, exemplifies a balanced, healthier alternative to modern processed snacks, aligning with dietary guidelines for reducing chronic disease risks [26].

This study assessed the nutritional value of snacks and desserts in Guizhou by analyzing FAs and sugar content. Rice-based foods, especially those made traditionally, like RT, have lower fat and sugar content, and a healthier FA profile. The study highlights the benefits of traditional cooking methods, particularly steaming, in preserving nutrients. This study innovatively investigated local snacks, which may provide scientific evidence for healthier dietary choices and promote traditional foods. However, certain limitations existed in this study. The first was the design of study on energy-based nutritional indicators (e.g., FA and sugar content), with less attention paid to non-energy-based nutritional indicators such as vitamins and minerals. Second, with a relatively smaller sample size, only 46 types of Guizhou snacks were included in the analysis. Future research should be performed by considering a wider variety of traditional snacks from different regions based on the expanded sample size. In this way, it may contribute to identifying more healthy options on snacks and provide a more comprehensive understanding of their nutritional value.

## Conclusions

This study assesses snacks and desserts in Guizhou province basing on FAs profile and sugar content. This study highlights the significant nutritional benefits of rice-based foods compared to those made from cream and wheat, particularly in terms of cardiovascular health. Rice products demonstrated lower levels of total FA and TFA, reduced AI and TI, and higher PUFA/SFA, indicating a lower risk of cardiovascular diseases. Traditional cooking methods, especially steaming, can effectively preserve the nutritional integrity of rice, further enhancing its health benefits. All together, findings in the present study support the promotion of rice-based foods, such as RT, as healthy dietary choices. It may provide a solid foundation for the development of local standards for RT and underscore the importance of incorporating traditional cooking methods into modern diets to improve overall health outcomes. To better integrate RT into a balanced diet, practical guidelines should be considered. For instance, RT can serve as a healthier snack alternative to high-fat and high-sugar options such as potato chips and cream cakes, due to its lower fat and calorie content. For individuals with diabetes or those aiming for weight management, RT can be an ideal choice for satisfying cravings without compromising dietary goals. Future research can be planned based on broader sampling of traditional Guizhou cuisine, thus supplying more profound implications for the development of the traditional food industry and cultural dissemination. Additionally, further studies should explore the role of RT and similar traditional rice products in diverse dietary patterns, providing evidence-based recommendations for their optimal incorporation into daily diets.

## Supporting information

S1 File This is the data for Fig 1. (PDF)

S2 File This is the data for Fig 2. (PDF)

S3 File This is the data for Fig 3. (PDF)

S4 File This is the data for Fig 4. (PDF)

S5 File This is the data for Fig 5. (PDF)

S6 File This is the data for Fig 6. (PDF)

S7 File Original raw data. This is the raw data for this manuscript.

## References

[1] Barnes JB, Smith JC, Ross KE, Whiley H. Performing food safety inspections. Food Control. 2024;160:110329. doi: 10.1016/j.foodcont.2024.110329

[2] Merz B, Temme E, Alexiou H, Beulens JWJ, Buyken AE, Bohn T, et al. Nutri-score 2023 update. Nat Food. 2024;5(2):102–10. doi: 10.1038/s43016-024-00920-3 38356074

[3] Zhang X, Huang T, Gao Y, Cai Y, Liu J, Ramachandraiah K, et al. Functional modification engineering of metal–organic frameworks for the contaminants detection in food. Coordination Chem Rev. 2024;516:215990. doi: 10.1016/j.ccr.2024.215990

[4] Li M, Li L, Tao X, Xie Z, Xie Q, Yuan J. Boosting healthiness exposure in category-constrained meal recommendation using nutritional standards. ACM Trans Intell Syst Technol. 2024;15(4):1–28. doi: 10.1145/3643859

[5] de Carvalho CCCR, Caramujo MJ. The various roles of fatty acids. Molecules. 2018;23(10):2583. doi: 10.3390/molecules23102583 30304860 PMC6222795

[6] Rybicka I, Gliszczyńska-Świgło A. Sugars in dairy products of different flavours. Int Dairy J. 2021;114:104933. doi: 10.1016/j.idairyj.2020.104933

[7] Bermingham KM, May A, Asnicar F, Capdevila J, Leeming ER, Franks PW, et al. Snack quality and snack timing are associated with cardiometabolic blood markers: the ZOE PREDICT study. Eur J Nutr. 2024;63(1):121–33. doi: 10.1007/s00394-023-03241-6 37709944 PMC10799113

[8] Mela DJ, Woolner EM. Perspective: total, added, or free? what kind of sugars should we be talking about? Adv Nutr. 2018;9(2):63–9. doi: 10.1093/advances/nmx020 29659689 PMC5916432

[9] Sędzikowska A, Szablewski L. Human glucose transporters in renal glucose homeostasis. Int J Mol Sci. 2021;22(24):13522. doi: 10.3390/ijms222413522 34948317 PMC8708129

[10] Thaiss CA, Levy M, Grosheva I, Zheng D, Soffer E, Blacher E, et al. Hyperglycemia drives intestinal barrier dysfunction and risk for enteric infection. Science. 2018;359(6382):1376–83. doi: 10.1126/science.aar3318 29519916

[11] Catanzaro R, Sciuto M, Marotta F. Lactose intolerance: an update on its pathogenesis, diagnosis, and treatment. Nutr Res. 2021;89:23–34. doi: 10.1016/j.nutres.2021.02.003 33887513

[12] Rosen ED, Spiegelman BM. What we talk about when we talk about fat. Cell. 2014;156(1–2):20–44. doi: 10.1016/j.cell.2013.12.012 24439368 PMC3934003

[13] Wang L, Chen Y, Yang Y, Xiao N, Lai C. Oils with different degree of saturation: effects on ileal digestibility of fat and corresponding additivity and bacterial community in growing pigs. J Anim Sci Biotechnol. 2024;15(1):21. doi: 10.1186/s40104-023-00990-6 38326917 PMC10848516

[14] Chen JQ. Examining the relationship between urbanization and food security in China. Impacts on Accessibilty, Affordability, and Food Quality. 2024.

[15] Agbaga M-P, Ahmad M. Emerging insights into the function of very long chain fatty acids at cerebellar synapses. Neural Regen Res. 2025;20(6):1709–10. doi: 10.4103/NRR.NRR-D-24-00436 39104105 PMC11688556

[16] National Health Commission of the People’s Republic of China. National standards for food safety: Determination of fatty acids in food. Beijing, China. National Health Commission: GB 5009.168-2016. 1st ed.; 2016.

[17] National Health Commission of the People’s Republic of China. National standards for food safety: Determination of sugars in food. Beijing, China: National Health Commission: GB 5009.8-2023. 1st ed; 2023.

[18] Dal Bosco A, Cavallo M, Menchetti L, Angelucci E, Cartoni Mancinelli A, Vaudo G, et al. The healthy fatty index allows for deeper insights into the lipid composition of foods of animal origin when compared with the atherogenic and thrombogenicity indexes. Foods. 2024;13(10):1568. doi: 10.3390/foods13101568 38790868 PMC11120502

[19] Mazur M, Przytuła A, Szymańska M, Popiołek-Kalisz J. Dietary strategies for cardiovascular disease risk factors prevention. Curr Probl Cardiol. 2024;49(9):102746. doi: 10.1016/j.cpcardiol.2024.102746 39002618

[20] Covaciu F-D, Feher I, Cristea G, Dehelean A. Nutritional quality and safety assessment of pork meat cuts from Romania: fatty acids and elemental profile. Foods. 2024;13(5):804. doi: 10.3390/foods13050804 38472917 PMC10930826

[21] Chaudhari P, Tamrakar N, Singh L, Tandon A, Sharma D. Rice nutritional and medicinal properties: a review article. J Pharmacog Phytochem. 7:150–6.

[22] Large JF, Madigan C, Pradeilles R, Markey O, Boxer B, Rousham EK. Impact of unhealthy food and beverage consumption on children’s risk of dental caries: a systematic review. Nutr Rev. 2024;82(11):1539–55. doi: 10.1093/nutrit/nuad147 38086176 PMC11465133

[23] Zhang Y, Jing L, Xu X, Ma T, Dong J. Dietary fatty acids intake and all-cause and cardiovascular mortality in patients on peritoneal dialysis. Clin Nutr. 2023;42(11):2188–97. doi: 10.1016/j.clnu.2023.09.002 37797355

[24] Mwale T, Rahman MM, Mondal D. Risk and benefit of different cooking methods on essential elements and Arsenic in rice. Int J Environ Res Public Health. 2018;15(6):1056. doi: 10.3390/ijerph15061056 29882885 PMC6025416

[25] Chen J, Liu H. Nutritional indices for assessing fatty acids: a mini-review. Int J Mol Sci. 2020;21(16):5695. doi: 10.3390/ijms21165695 32784511 PMC7460856

[26] Chinese Nutrition Society. China dietary guidelines. 1st ed. People’s Medical Publishing House.

[27] Rabail R, Aadil RM, Sahar A, Zia MA. Nutritional and physicochemical analysis of edible oil blend with improved ratios of cardioprotective nutritional indices and physicochemical properties. Food Measure. 2024;18(5):3584–94. doi: 10.1007/s11694-024-02429-6

[28] Yu L, Turner MS, Fitzgerald M, Stokes JR, Witt T. Review of the effects of different processing technologies on cooked and convenience rice quality. Trends Food Sci Technol. 2017;59:124–38. doi: 10.1016/j.tifs.2016.11.009

[29] Zohoun EV, Tang EN, Soumanou MM, Manful J, Akissoe NH, Bigoga J, et al. Physicochemical and nutritional properties of rice as affected by parboiling steaming time at atmospheric pressure and variety. Food Sci Nutr. 2018;6(3):638–52. doi: 10.1002/fsn3.600 29876115 PMC5980200

[30] Go M, Nm E. Analysis of utilization of traditional medicine for the treatment of malaria among rural farmers in Abia State, Nigeria. Int J Environ Agric Biotechnol. 2019;4(2):374–8. doi: 10.22161/ijeab/4.2.17

[31] Mai LaB. Analysis of the main differences and integration strategies between Chinese and Western dim sum. Food Safety Guide. 2025:160–2. doi: 10.16043/j.cnki.cfs.2025.05.057

[32] Huang Q, Zhao Y, Zhou C, Wang S, Ding J, Deng T. Study on the impact of chinese and western processing techniques on sausage quality. China Condiment. 105–8.

[33] Song Z. Minority traditional culture and the consciousness of the chinese national community: a case study of the food culture of miao and dong ethnic groups in Guizhou. Guizhou Ethnic Studies. 122–129. doi: 10.13965/j.cnki.gzmzyj10026959.2021.01.017

[34] Chen H, Zhang X. Evaluating the nutritional quality of ethnic foods: Methodological challenges and opportunities. J Ethnic Food. 2022;6:145–60.

[35] Yasmeen B, Fischer F. Food choices of contemporary cuisine and traditional foods: effects on family ties. Nutrients. 2024;16(23):4126. doi: 10.3390/nu16234126 39683520 PMC11644626

[36] Wardana AA, Setiarto RHB. Unveiling the cultural significance and development of “wajik”, a traditional Javanese food. J Ethn Food. 2024;11(1). doi: 10.1186/s42779-024-00237-3

[37] Hadian Z, Mousavi Khaneghah A. Sugar, fat, saturated and trans fatty acid contents in Iranian cereal-based baked products. Food Sci Technol. 2022;42. doi: 10.1590/fst.26724

[38] Barakat H, Aljutaily T, Khalifa I, Almutairi AS, Aljumayi H. nutritional properties of innovatively prepared plant-based vegan snack. Processes. 2024;12(12):2720. doi: 10.3390/pr12122720

