# Supplementary material for: Health assessment of snacks and desserts in Guizhou Province: Analysis of fatty acids and sugar content
Source: PLoS One. 2025 Jun 2;20(6):e0321857. doi: 10.1371/journal.pone.0321857 (PMC12129230; doi:10.1371/journal.pone.0321857)
Supplement: S1 File — (PDF) [file pone.0321857.s001.pdf]

		Dessert/ snacks Name	abbreviation	processing method	main component	Crude Fat (g/100g)	Total Fatty Acids (g/100g)	TFA(g/100g)
YP13		Tiramisu	TI	Steaming	**cream**	27.88	27.5883	0.3756
YP19		Semi-cooked Cheesecake	SCC	Baking	**cream**	26.97	20.2911	0.5396
YP22		Black Forest Cake	BFC	Steaming	**cream**	25.82	22.6939	0.0566
YP24		Xue Meiniang	XMN	Steaming	**cream**	17.78	14.1538	0.221
YP27		Lime Cake	LC	Steaming	**cream**	26.30	23.6715	0.4278
YP31		Blueberry Cake	BC	Steaming	**cream**	38.27	30.6442	0.8989
YP33		Taro Cream Cake	TCC	Baking	**cream**	37.80	34.6787	0.9433
YP38		Durian Mille-Feuille	DMF	Baking	**cream**	17.11	16.0871	0.2086
YP47		Strawberry Flavored Ice Cream Mooncake	SFICM	Steaming	cream	19.65	16.7301	0.2207
YP48		Rum and Grape Flavored Ice Cream Mooncake	RGICM	Baking	cream	26.46	19.2115	0.338
YP49		Vanilla and Macadamia Nut Flavored Ice Cream Mooncake	VMNFICM	Steaming	cream	28.11	24.2159	0.3652
YP12		Mochi	MO	Baking	**rice**	19.00	17.4854	0.1008
YP15	Tradition	Crab Roe Crisps	CRC	Frying	Rice	45.81	41.2443	0.072
YP17		Red Bean Bun	RBB	Baking	**rice**	14.23	11.3702	0.064
YP25	Tradition	Sesame crisp	SC	Frying	**rice**	49.01	45.9475	0.1371

YP28	Tradition	Niu Dagun	ND	Steaming	Rice	9.45	8.4171	0
YP3		Taosu	TS	Frying	**rice**	38.43	35.3298	0.047
YP34	Tradition	Purple Rice Cake	PRC	Baking	Rice	26.92	23.8098	0.6063
YP42	Tradition	Cocont and Apple Mooncake with Litsea Cubeba Flavor	CAMLC	Steaming	rice	6.65	5.3423	0.0328
YP43		Golden Salad Creamy Yolk and Nut Mooncake	GSCYNM	Steaming	rice	14.50	12.3028	0.0487
YP44		Vanilla Flavored Coffee Mooncake	VFCM	Steaming	rice	13.21	9.1885	0.0662
YP45		Tangerine and Pomelo Flavored Coffee Mooncake	TPFM	Steaming	rice	7.69	6.8232	0.0496
YP46		Cocoa Flavored Coffee Mooncake	CFCM	Steaming	rice	13.09	9.0996	0.0557
YP50	Tradition	Rice Tofu	RT	Steaming	Rice	5.98	5.1913	0.0163
YP51	Tradition	Cotton Grass Rice Cake	CGRC	Steaming	Rice	7.32	5.4386	0.0223
YP52	Tradition	RICE CAKE	RC	Baking	RICE	16.71	15.4803	0.0842
YP1		Caramel Treats	CT	Frying	**Wheat**	29.27	27.2866	0.0286
YP10		Hand-torn Bread	HTB	Baking	**Wheat**	18.43	15.6912	0.0461
YP11		Dried Meat Floss Bread	DMFB	Baking	**Wheat**	18.64	17.9762	0.0302

YP14		Puff Pastry	PP	Baking	Wheat	35.20	29.74844	0.2105
YP16		Pineapple Bun	PB	Baking	**Wheat**	15.38	12.6386	0.0642
YP18		Egg Roll	ER	Baking	**Wheat**	29.64	23.8207	0.5352
YP2		Cookies	CK	Baking	**Wheat**	41.24	39.106	0.3792
YP20		Dried Meat Floss Bun	DMFb	Baking	**Wheat**	34.13	27.4947	0.0194
YP23	Tradition	Zunyi Cake	ZC	Baking	**Wheat**	8.69	6.106	0
YP26	Tradition	Yuanzu Pineapple Cake	YPC	Baking	Wheat	16.40	16.2417	0.505
YP35	Tradition	Cui Bobo Cake	CBC	Baking	Wheat	42.85	38.0716	1.1007
YP36		Cake Roll	CR	Baking	Wheat	29.31	28.1201	0.2891
YP37		Strawberry Magic Wand	SMW	Baking	Wheat	15.56	12.7266	0.2313
YP4		Egg and Milk Toast	EMT	Baking	Wheat	11.89	9.9918	0.2524
YP40	Tradition	Roasted Ham Mooncake	RHM	Baking	**Wheat**	36.81	31.389	0.1053
YP41		Muffin Cake	MC	Baking	**Wheat**	27.87	21.5784	0.1114
YP5		Sandwich	SD	Baking	**Wheat**	16.58	15.4986	0.084
YP7		Coconut Cream	CC	Baking	**Wheat**	33.20	32.0066	0.1461
YP8		Milk Flavored Wafers	MFW	Baking	Wheat	26.34	24.8469	0.0495
YP9		Donuts	DN	Frying	Wheat	28.06	23.3371	0.145

MUFA/ (g/100g)	PUFA/SFA	n-3PUFA/n	AI	TI	Crude Fat (g/100g)	Total Fatty Acids (g/100g)	TFA(g/100g)
8.1398	0.153036663	0.07	1.45	2.01	26.56	22.72	0.4178
2.0988	0.186472403	0.10	2.31	3.64	6.95	6.36	0.2802
4.3628	0.281921998	0.08	1.92	1.64			
3.4335	0.052878147	0.11	2.31	3.15			
6.5687	0.138312246	0.04	1.33	2.44			
8.6175	0.050080568	0.15	1.65	2.92			
9.3801	0.136739847	0.13	1.44	2.14			
3.4516	0.145401804	0.11	2.30	2.17			
4.3205	0.230903518	0.08	1.27	1.84			
5.7168	0.28958182	0.09	0.79	1.69			
9.427	0.242096687	0.08	0.63	1.41			
7.2606	2.682887296	0.09	0.24	0.21	19.20	16.83	0.0935
18.4907	0.274618655	0.01	0.14	1.43	14.28	13.56	0.1459
4.0543	0.553797468	0.09	0.36	1.08			
15.8634	3.169082594	0.07	0.06	0.29			

2.7189	1.260472866	0.25	0.10	0.51			
15.7688	0.295919652	0.02	0.23	1.42			
6.5937	0.178918464	0.12	1.26	1.89			
2.1161	0.815429633	0.16	0.71	0.29			
6.0291	0.636546237	0.19	0.22	0.58			
2.8909	2.22111401	0.11	0.13	0.34			
1.6679	2.441914808	0.12	0.14	0.34			
3.1402	0.825293271	0.10	0.29	0.74			
3.1061	8.482492042	0.25	0.04	0.05			
2.7677	0.685854952	0.12	0.18	0.66			
5.0609	0.889438886	0.98	9.2007	9.1165			
10.5741	2.199115637	0.05	0.19	0.39	25.77	22.68	0.2167
5.4609	0.303771012	0.05	0.42	1.62	9.97	9.18	0.2602
5.2659	2.141370703	0.07	0.11	0.42			

7.3455	0.170483649	0.01	1.65	2.30
4.3506	0.269180117	0.05	0.49	1.69
10.0474	0.26299139	0.01	0.22	1.50
13.9089	0.448408866	0.07	0.33	1.24
8.1046	2.944524686	0.09	0.07	0.31
3.6617	3.439338903	0.06	0.10	0.16
4.0449	0.108537982	0.16	1.70	2.41
10.7915	0.120995578	0.13	1.42	2.29
7.5643	0.355163662	0.09	0.97	1.36
3.7052	0.211251343	0.10	1.04	1.86
2.5986	0.128267737	0.12	1.47	2.42
14.6149	0.329294386	0.04	0.33	1.26
6.4747	2.227838092	0.09	0.07	0.42
5.0617	0.839717262	0.07	0.30	0.85
8.9302	0.192369299	0.06	1.14	2.00
15.0924	1.175304402	0.06	0.30	0.21
7.8586	0.268677513	0.05	0.55	1.73

MUFA/ (g/100g)	PUFA/SFA	n-3PUFA/n-6I	AI	TI		
5.96	0.17	0.09	1.58	2.28	mean	cream
2.62	0.08	0.03	0.59	0.69	std	
6.50	1.69	0.18	0.89	1.26	mean	Rice
5.56	2.11	0.23	2.32	2.24	std	

7.77	0.91	0.07	0.64	1.32	mean	Wheat
3.76	1.06	0.04	0.57	0.79	std	
