# Supplementary material for: Health assessment of snacks and desserts in Guizhou Province: Analysis of fatty acids and sugar content
Source: PLoS One. 2025 Jun 2;20(6):e0321857. doi: 10.1371/journal.pone.0321857 (PMC12129230; doi:10.1371/journal.pone.0321857)
Supplement: S2 File — (PDF) [file pone.0321857.s002.pdf]

sample number		Dessert/ snacks Name	abbreviation	processing method	main component	Glucose (g	Fructose (g
YP13		Tiramisu	TI	Steaming	**cream**	0.360526839	—
YP19		Semi-cooked Cheesecake	SCC	Baking	**cream**	0.248787276	—
YP22		Black Forest Cake	BFC	Steaming	**cream**	—	—
YP24		Xue Meiniang	XMN	Steaming	**cream**	1.124141717	—
YP27		Lime Cake	LC	Steaming	**cream**	—	0.379830677
YP31		Blueberry Cake	BC	Steaming	**cream**	—	0.236207585
YP33		Taro Cream Cake	TCC	Baking	**cream**	—	—
YP38		Durian Mille-Feuille	DMF	Baking	**cream**	0.286025896	—
YP47		Strawberry Flavored Ice Cream Mooncake	SFICM	Steaming	cream	0.24502994	—
YP48		Rum and Grape Flavored Ice Cream Mooncake	RGICM	Baking	cream	—	—
YP49		Vanilla and Macadamia Nut Flavored Ice Cream Mooncake	VMNFICM	Steaming	cream	—	—
YP12		Mochi	MO	Baking	**rice**	0.199173307	0.281
YP15	Tradition	Crab Roe Crisps	CRC	Frying	Rice	—	0.220
YP17		Red Bean Bun	RBB	Baking	**rice**	—	0.260
YP25	Tradition	Sesame crisp	SC	Frying	**rice**	—	0.19998008

YP28	Tradition	Niu Dagun	ND	Steaming	Rice	0.648	—
YP3		Taosu	TS	Frying	**rice**	—	—
YP34	Tradition	Purple Rice Cake	PRC	Baking	Rice	0.263	0.146523904
YP42	Tradition	Cocont and Apple Mooncake with Litsea Cubeba Flavor	CAMLC	Steaming	rice	0.160	—
YP43		Golden Salad Creamy Yolk and Nut Mooncake	GSCYNM	Steaming	rice	—	—
YP44		Vanilla Flavored Coffee Mooncake	VFCM	Steaming	rice	—	—
YP45		Tangerine and Pomelo Flavored Coffee Mooncake	TPFM	Steaming	rice	0.213	—
YP46		Cocoa Flavored Coffee Mooncake	CFCM	Steaming	rice	0.403	—
YP50	Tradition	Rice Tofu	RT	Steaming	Rice	0.296147705	0.916646707
YP51	Tradition	Cotton Grass Rice Cake	CGRC	Steaming	Rice	0.225199601	—
YP52	Tradition	RICE CAKE	RC	Baking	RICE	0.890	0.331
YP1		Caramel Treats	CT	Frying	**Wheat**	0.181	0.278403194
YP10		Hand-torn Bread	HTB	Baking	**Wheat**	1.503642715	1.253
YP11		Dried Meat Floss Bread	DMFB	Baking	**Wheat**	0.848	0.642

YP14		Puff Pastry	PP	Baking	Wheat	0.252	—
YP16		Pineapple Bun	PB	Baking	**Wheat**	1.24502988	0.989203187
YP18		Egg Roll	ER	Baking	**Wheat**	—	—
YP2		Cookies	CK	Baking	**Wheat**	—	—
YP20		Dried Meat Floss Bun	DMFb	Baking	**Wheat**	—	2.325
YP23	Traditior	Zunyi Cake	ZC	Baking	**Wheat**	0.443	0.564720559
YP26	Traditior	Yuanzu Pineapple Cake	YPC	Baking	Wheat	0.444	0.514
YP35	Traditior	Cui Bobo Cake	CBC	Baking	Wheat	—	—
YP36		Cake Roll	CR	Baking	Wheat	0.288	—
YP37		Strawberry Magic Wand	SMW	Baking	Wheat	1.051586826	—
YP4		Egg and Milk Toast	EMT	Baking	Wheat	2.187	1.357
YP40	Traditior	Roasted Ham Mooncake	RHM	Baking	**Wheat**	0.184	—
YP41		Muffin Cake	MC	Baking	**Wheat**	—	—
YP5		Sandwich	SD	Baking	**Wheat**	0.900	0.311
YP7		Coconut Cream	CC	Baking	**Wheat**	0.793	0.606
YP8		Milk Flavored Wafers	MFW	Baking	Wheat	—	—
YP9		Donuts	DN	Frying	Wheat	0.603	0.512892644

crose (g/100	Maltose (g/	Lactose(g/1	The total s	Glucose (g/	Fructose (g/
—	—	0.235	0.595	0.452902334	0.308019131
0.207	—	—	0.456	0.37809297	0.101556863
0.572	—	—	0.572		
—	5.923	0.510548902	7.557		
0.293	0.31189243	—	0.985		
0.588173653	0.211	—	1.035		
—	—	—	—		
—	—	0.13624502	0.422		
1.079590818	0.504570858	—	1.829191617		
0.641227545	0.452714571	—	1.093942116		
0.754233068	0.469013944	—	1.223247012		
6.863	1.122	0.193237052	8.659	0.366298225	0.336362199
0.225888224	—	—	0.445	0.245931498	0.262668135
2.311	0.54508982	—	3.116		
1.47625498	—	—	1.67623506		

—	0.291341948	—	0.939		
3.298	0.110	—	3.408		
—	—	—	0.409		
0.895	1.501	—	2.557		
0.552	—	—	0.552		
0.824	—	—	0.824		
1.22373506	—	—	1.437		
3.794383698	—	—	4.197		
—	—	—	1.212794411		
—	—	—	0.225199601		
—	0.371	0.463	2.055		
0.570	1.873	—	2.903	0.78014322	0.850367449
2.059	0.695369261	0.357305389	5.869	0.575532776	0.603572753
2.327	0.389	—	4.206		

—	—	0.213542914	0.465
3.388	0.519342629	0.443386454	6.585
8.439561753	0.183	0.103	8.725
1.964	—	—	1.964
—	0.416516966	—	2.742
26.39447106	—	—	27.402
0.63750499	—	—	1.596
0.290	—	—	0.290
—	—	—	0.288
—	0.678463074	0.272115768	2.002
0.944	1.624	1.341	7.452
2.816	—	—	3.000
0.586	—	—	0.586
—	0.381	0.433	2.025
1.857	0.553	—	3.809
7.298496016	0.456085657	—	7.755
1.281421471	0.838807157	1.147	4.383

crose (g/100	Maltose (g/	Lactose(g/1	The total sugar (g/100g)		
0.590844802	1.311944143	0.2939048	1.57698428	mean	cream
0.289855753	2.261494088	0.193997969	2.144589134	std	
2.146388579	0.656767726	0.327876011	2.114097573	mean	Rice
2.031995902	0.539555245	0.190408242	2.1753772	std	

4.056798631	0.717296952	0.538780917	4.702291862	mean	Wheat
6.624398337	0.513955618	0.452550082	5.937524367	std	
