# Supplementary material for: Health assessment of snacks and desserts in Guizhou Province: Analysis of fatty acids and sugar content
Source: PLoS One. 2025 Jun 2;20(6):e0321857. doi: 10.1371/journal.pone.0321857 (PMC12129230; doi:10.1371/journal.pone.0321857)
Supplement: S3 File — (PDF) [file pone.0321857.s003.pdf]

		Dessert/s	abbreviation		processin	main comp	Crude Fat	Total Fat
YP12		Mochi	MO	1	Baking	rice	19	17.4854
YP17		Red Bean	RBB	2	Baking	rice	14.23	11.3702
YP34	Tradition	Purple Ri	PRC	3	Baking	Rice	26.92	23.8098
YP52	Tradition	RICE CAKE	RC	4	Baking	RICE	16.71	15.4803
YP15	Tradition	Crab Roe	CRC	5	Frying	Rice	45.81	41.2443
YP25	Tradition	Sesame cr	SC	6	Frying	rice	49.01	45.9475
YP3		Taosu	TS	7	Frying	rice	38.43	35.3298
YP28	Tradition	Niu Dagun	ND	8	Steaming	Rice	9.45	8.4171
YP42	Tradition	Cocont an	CAMLC	9	Steaming	rice	6.65	5.3423
YP43		Golden Sa	GSCYNM	10	Steaming	rice	14.5	12.3028
YP44		Vanilla F	VFCM	11	Steaming	rice	13.21	9.1885
YP45		Tangerine	TPFM	12	Steaming	rice	7.69	6.8232
YP46		Cocoa Fla	CFCM	13	Steaming	rice	13.09	9.0996
YP50	Tradition	Rice Tofu	RT	14	Steaming	Rice	5.98	5.1913
YP51	Tradition	Cotton Gr	CGRC	15	Steaming	Rice	7.32	5.4386

TFA(g/10C	MUFA/ (g/	PUFA/SFA	n-3PUFA/n-6PU	AI	TI	Crude Fat (g
0.1008	7.2606	2.682887296	0.092620029	0.235439286	0.208560693	19.22
0.064	4.0543	0.553797468	0.090730361	0.356104957	1.075309952	5.49
0.6063	6.5937	0.178918464	0.115199112	1.259751263	1.890139746	
0.0842	5.0609	0.889438886	0.979042426	9.2007	9.1165	
0.072	18.4907	0.274618655	0.012014616	0.140845552	1.42828405	44.42
0.1371	15.8634	3.169082594	0.067311058	0.061613932	0.289484225	5.43
0.047	15.7688	0.295919652	0.024261047	0.226048281	1.424531232	
0	2.7189	1.260472866	0.254253345	0.103743025	0.506747489	9.74
0.0328	2.1161	0.815429633	0.155121562	0.707954673	0.292217515	3.38
0.0487	6.0291	0.636546237	0.192085979	0.217396951	0.575259128	
0.0662	2.8909	2.22111401	0.113918531	0.130865153	0.33741879	
0.0496	1.6679	2.441914808	0.118467325	0.139801705	0.340136841	
0.0557	3.1402	0.825293271	0.103805661	0.285824464	0.735418121	
0.0163	3.1061	8.482492042	0.252551706	0.042784729	0.049585406	
0.0223	2.7677	0.685854952	0.124611882	0.182705031	0.660840356	

Total Fatty	TFA(g/100g)	MUFA/ (g/100	PUFA/SFA	n-3PUFA/n-6P	AI
17.04	0.2138	5.74	1.08	0.32	2.76
5.18	0.2621	1.45	1.11	0.44	4.32
40.84	0.0854	16.71	1.25	0.03	0.14
5.32	0.0465	1.54	1.67	0.03	0.08
7.73	0.0365	3.05	2.17	0.16	0.23
2.50	0.0224	1.30	2.64	0.06	0.21

TI

3.07	mean	baking
4.09	std	
1.05	mean	Frying
0.66	std	
0.44	mean	Steaming
0.22	std	
