# Supplementary material for: Health assessment of snacks and desserts in Guizhou Province: Analysis of fatty acids and sugar content
Source: PLoS One. 2025 Jun 2;20(6):e0321857. doi: 10.1371/journal.pone.0321857 (PMC12129230; doi:10.1371/journal.pone.0321857)
Supplement: S4 File — (PDF) [file pone.0321857.s004.pdf]

sample number		Dessert/s	abbreviation		processin	main comp	Glucose (g/
YP12		Mochi	MO	1	Baking	rice	0.199173307
YP17		Red Bean	RBB	2	Baking	rice	—
YP34	Tradition	Purple Ri	PRC	3	Baking	Rice	0.262778884
YP52	Tradition	RICE CAKE	RC	4	Baking	RICE	0.889730539
YP15	Tradition	Crab Roe	CRC	5	Frying	Rice	—
YP25	Tradition	Sesame cr	SC	6	Frying	rice	—
YP3		Taosu	TS	7	Frying	rice	—
YP28	Tradition	Niu Dagun	ND	8	Steaming	Rice	0.647673956
YP42	Tradition	Cocont an	CAMLC	9	Steaming	rice	0.160397614
YP43		Golden Sa	GSCYNM	10	Steaming	rice	—
YP44		Vanilla F	VFCM	11	Steaming	rice	—
YP45		Tangerine	TPFM	12	Steaming	rice	0.212958167
YP46		Cocoa Fla	CFCM	13	Steaming	rice	0.402624254
YP50	Tradition	Rice Tofu	RT	14	Steaming	Rice	0.296147705
YP51	Tradition	Cotton Gr	CGRC	15	Steaming	Rice	0.225199601

Fructose (g/:	Sucrose (g/:	Maltose (g/1(	Lactose(g/10(	The total su{	Glucose (g/
0.28126494	6.863077689	1.121822709	0.193237052	8.658575697	0.451
0.259640719	2.311087824	0.54508982	—	3.115818363	0.382
0.146523904	—	—	—	0.409302789	
0.330938124	—	0.371497006	0.46251497	2.054680639	
0.219540918	0.225888224	—	—	0.445429142	0
0.19998008	1.47625498	—	—	1.67623506	0
—	3.298071571	0.109821074	—	3.407892644	
—	—	0.291341948	—	0.939015905	0.324
—	0.895119284	1.501033797	—	2.556550696	0.179
—	0.551856287	—	—	0.551856287	
—	0.824411178	—	—	0.824411178	
—	1.22373506	—	—	1.436693227	
—	3.794383698	—	—	4.197007952	
0.916646707	—	—	—	1.212794411	
—	—	—	—	0.225199601	

Fructose (g/	Sucrose (g/	Maltose (g/1	Lactose(g/10	The total sugar (g/100g)		
0.255	4.587	0.679	0.328	3.560	mean	baking
0.078	3.219	0.393	0.190	3.577	std	
0.210	1.667	0.110	0	1.843	mean	Frying
0.014	1.545	0	0	1.488	std	
0.917	1.458	0.896	0	1.493	mean	Steaming
0	1.328	0.855	0	1.297	std	
