# Supplementary material for: Health assessment of snacks and desserts in Guizhou Province: Analysis of fatty acids and sugar content
Source: PLoS One. 2025 Jun 2;20(6):e0321857. doi: 10.1371/journal.pone.0321857 (PMC12129230; doi:10.1371/journal.pone.0321857)
Supplement: S5 File — (PDF) [file pone.0321857.s005.pdf]

		Dessert/s	abbreviat	processin	main comp	Crude Fa	Total Fa	TFA(g/100
YP28	Tradition	Niu Dagun	ND	Steaming	Rice	9.45	8.42	0
YP42	Tradition	Cocont an	CAMLC	Steaming	rice	6.65	5.34	0.0328
YP50	Tradition	Rice Tofu	RT	Steaming	Rice	5.98	5.19	0.0163
YP51	Tradition	Cotton Gr	CGRC	Steaming	Rice	7.32	5.44	0.0223
YP43		Golden Sa	GSCYNM	Steaming	rice	14.50	12.30	0.0487
YP44		Vanilla F	VFCM	Steaming	rice	13.21	9.19	0.0662
YP45		Tangerine	TPFM	Steaming	rice	7.69	6.82	0.0496
YP46		Cocoa Fla	CFCM	Steaming	rice	13.09	9.10	0.0557

MUFA/ (g	PUFA/SFA	n-3PUFA/n-6I	AI	TI	Crude Fa	Total Fa	TFA(g/10
2.72	1.26	0.25	0.10	0.51	7.35	6.10	0.0179
2.12	0.82	0.16	0.71	0.29	1.50	1.55	0.0137
3.11	8.48	0.25	0.04	0.05			
2.77	0.69	0.12	0.18	0.66			
6.03	0.64	0.19	0.22	0.58	12.12	9.35	0.0551
2.89	2.22	0.11	0.13	0.34	3.02	2.25	0.0081
1.67	2.44	0.12	0.14	0.34			
3.14	0.83	0.10	0.29	0.74			

MUFA/ (g	PUFA/SFA	n-3PUFA/	AI	TI		
2.68	2.81	0.20	0.26	0.38	mean	Traditional
0.41	3.79	0.07	0.30	0.27	std	
3.43	1.53	0.13	0.19	0.50	mean	common
1.85	0.93	0.04	0.07	0.19	std	
