# Supplementary material for: Health assessment of snacks and desserts in Guizhou Province: Analysis of fatty acids and sugar content
Source: PLoS One. 2025 Jun 2;20(6):e0321857. doi: 10.1371/journal.pone.0321857 (PMC12129230; doi:10.1371/journal.pone.0321857)
Supplement: S6 File — (PDF) [file pone.0321857.s006.pdf]

| sample number | | | Dessert/snacks Name | abbreviat | processin | main comp |
|---|---|---|---|---|---|---|
| YP28 | 1 | Tradition | Niu Dagun | ND | Steaming | Rice |
| YP50 | 1 | Tradition | Rice Tofu | RT | Steaming | Rice |
| YP51 | 1 | Tradition | Cotton Grass Rice Cake | CGRC | Steaming | Rice |
| YP42 | 2 | Tradition | Cocont and Apple Mooncake w | CAMLC | Steaming | rice |
| YP43 | | | Golden Salad Creamy Yolk an | GSCYNM | Steaming | rice |
| YP44 | | | Vanilla Flavored Coffee Moo | VFCM | Steaming | rice |
| YP45 | | | Tangerine and Pomelo Flavor | TPFM | Steaming | rice |
| YP46 | | | Cocoa Flavored Coffee Moonc | CFCM | Steaming | rice |

| Glucose (g/[illegible] | Fructose (g/[illegible] | Sucrose (g/[illegible] | Maltose (g/1[illegible] | Lactose(g/10[illegible] | The total su[illegible] |
|---|---|---|---|---|---|
| 0.647673956 | — | — | 0.291341948 | — | 0.939015905 |
| 0.296147705 | 0.916646707 | — | — | — | 1.212794411 |
| 0.225199601 | — | — | — | — | 0.225199601 |
| 0.160397614 | — | 0.895119284 | 1.501033797 | — | 2.556550696 |
| — | — | 0.551856287 | — | — | 0.551856287 |
| — | — | 0.824411178 | — | — | 0.824411178 |
| 0.212958167 | — | 1.22373506 | — | — | 1.436693227 |
| 0.402624254 | — | 3.794383698 | — | — | 4.197007952 |

| Glucose (g/ | Fructose (g/ | Sucrose (g/ | Maltose (g/1 | Lactose(g/1( | The total sugar (g/1( | |
|---|---|---|---|---|---|---|
| 0.332 | 0.917 | 0.895 | 0.896 | 0 | 1.233 | mean |
| 0.217 | 0 | 0 | 0.855 | 0 | 0.975 | std |
| | | | | | | |
| 0.308 | 0 | 1.599 | 0 | 0 | 1.752 | mean |
| 0.134 | 0 | 1.490 | 0 | 0 | 1.671 | std |

)0g)
Traditional

common
