# Supplementary material for: Health assessment of snacks and desserts in Guizhou Province: Analysis of fatty acids and sugar content
Source: PLoS One. 2025 Jun 2;20(6):e0321857. doi: 10.1371/journal.pone.0321857 (PMC12129230; doi:10.1371/journal.pone.0321857)
Supplement: S7 File — This is the raw data for this manuscript. [file pone.0321857.s007.pdf]

							SFA	SFA
		Dessert/ snacks Name	abbreviation	processing method	**main component**	Crude Fat (g/100g)	Methyl butyrate（C_4: 0）	Methyl hexanoate（C_6: 0）
YP1		Caramel Treats	CT	Frying	**Wheat**	29.27	0	0
YP10		Hand-torn Bread	HTB	Baking	**Wheat**	18.43	0.0375	0.0281
YP11		Dried Meat Floss Bread	DMFB	Baking	**Wheat**	18.64	0.0235	0.0182
YP12		Mochi	MO	Baking	**rice**	19.00	0.0693	0.0389
YP13		Tiramisu	TI	Steaming	**cream**	27.88	0.5754	0.3149
YP14		Puff Pastry	PP	Baking	Wheat	35.20	0.1166	0.1292
YP15	Tradition	Crab Roe Crisps	CRC	Frying	Rice	45.81	0	0
YP16		Pineapple Bun	PB	Baking	**Wheat**	15.38	0.112	0.0501
YP17		Red Bean Bun	RBB	Baking	**rice**	14.23	0.0669	0.0314
YP18		Egg Roll	ER	Baking	**Wheat**	29.64	0	0
YP19		Semi-cooked Cheesecake	SCC	Baking	**cream**	26.97	0.8049	0.3897
YP2		Cookies	CK	Baking	**Wheat**	41.24	0.2675	0.1304
YP20		Dried Meat Floss Bun	DMFb	Baking	**Wheat**	34.13	0.0142	0.0049
YP22		Black Forest Cake	BFC	Steaming	**cream**	25.82	0.0605	0.0568
YP23	Tradition	Zunyi Cake	ZC	Baking	**Wheat**	8.69	0	0
YP24		Xue Meiniang	XMN	Steaming	**cream**	17.78	0.5697	0.2482
YP25	Tradition	Sesame crisp	SC	Frying	**rice**	49.01	0	0
YP26	Tradition	Yuanzu Pineapple Cake	YPC	Baking	Wheat	16.40	0.9541	0.3865

YP27		Lime Cake	LC	Steaming	**cream**	26.30	1.2576	0.5082
YP28	Tradition	Niu Dagun	ND	Steaming	Rice	9.45	0	0
YP3		Taosu	TS	Frying	**rice**	38.43	0	0
YP31		Blueberry Cake	BC	Steaming	**cream**	38.27	1.5512	0.65
YP33		Taro Cream Cake	TCC	Baking	**cream**	37.80	1.777	0.7012
YP34	Tradition	Purple Rice Cake	PRC	Baking	Rice	26.92	0.925	0.3892
YP35	Tradition	Cui Bobo Cake	CBC	Baking	Wheat	42.85	1.8419	0.7617
YP36		Cake Roll	CR	Baking	Wheat	29.31	0.6473	0.3146
YP37		Strawberry Magic Wand	SMW	Baking	Wheat	15.56	0.4587	0.1918
YP38		Durian Mille-Feuille	DMF	Baking	**cream**	17.11	0.3153	0.1396
YP4		Egg and Milk Toast	EMT	Baking	Wheat	11.89	0.3111	0.1763
YP40	Tradition	Roasted Ham Mooncake	RHM	Baking	**Wheat**	36.81	0	0
YP41		Muffin Cake	MC	Baking	**Wheat**	27.87	0	0
YP42	Tradition	Cocont and Apple Mooncake with Litsea Cubeba Flavor	CAMLC	Steaming	rice	6.65	0.07	0.0431
YP43		Golden Salad Creamy Yolk and Nut Mooncake	GSCYNM	Steaming	rice	14.50	0.0839	0.0394
YP44		Vanilla Flavored Coffee Mooncake	VFCM	Steaming	rice	13.21	0.0398	0.0183

YP45		Tangerine and Pomelo Flavored Coffee Mooncake	TPFM	Steaming	rice	7.69	0.0354	0.0146
YP46		Cocoa Flavored Coffee Mooncake	CFCM	Steaming	rice	13.09	0.0358	0.0215
YP47		Strawberry Flavored Ice Cream Mooncake	SFICM	Steaming	cream	19.65	0.4028	0.1858
YP48		Rum and Grape Flavored Ice Cream Mooncake	RGICM	Baking	cream	26.46	0.4997	0.2359
YP49		Vanilla and Macadamia Nut Flavored Ice Cream Mooncake	VMNFICM	Steaming	cream	28.11	0.6677	0.2679
YP5		Sandwich	SD	Baking	**Wheat**	16.58	0.1229	0.0002
YP7		Coconut Cream	CC	Baking	**Wheat**	33.20	0.1906	0.1487
YP8		Milk Flavored Wafers	MFW	Baking	Wheat	26.34	0.0252	0.0367
YP9		Donuts	DN	Frying	Wheat	28.06	0.0551	0.0446
YP50	Tradition	Rice Tofu	RT	Steaming	Rice	5.98	0	0
YP51	Tradition	Cotton Grass Rice Cake	CGRC	Steaming	Rice	7.32	0	0
YP52	Tradition	RICE CAKE	RC	Baking	RICE	16.71	0.1329	0.0003

SFA	SFA	SFA	SFA	SFA	SFA	SFA	SFA	SFA
Methyl octanoate（C_8:0）	Methyl decanoate（C_{10}:0）	Methyl laurate（C_{12}:0）	Methyl tridecanoate（C_{13}:0）	Methyl myristate（C_{14}:0）	Methyl pentadecanoate（C_{15}:0）	Methyl palmitate（C_{16}:0）	Methyl heptadecanoate（C_{17}:0）	Methyl stearate（C_{18}:0）
0	0	0.0091	0	0.0692	0	3.9685	0.0163	0.7546
0.1131	0.0998	0.5872	0	0.4264	0.0173	5.4445	0.0211	0.9927
0.0232	0.0333	0.098	0	0.146	0.0163	2.5632	0.0176	0.8676
0.0565	0.0731	0.4338	0	0.3823	0.0258	0	0.0395	1.5001
0.2576	0.5091	2.804	0	2.589	0.1629	6.7938	0.103	2.3844
0.2899	0.4379	4.5489	0.0167	2.5961	0.085	7.2026	0.0726	2.6162
0.0191	0.0082	0.0997	0.1826	0.3268	0.0147	14.7162	0.0333	1.8879
0.0888	0.0988	0.439	0	0.4083	0.0237	4.2125	0.0264	0.9406
0.0279	0.0546	0.1318	0	0.2645	0.0204	2.8154	0.0224	1.1825
0.0337	0.0284	0.335	0.1742	0.265	0.093	8.1135	0.0214	1.4594
0.2214	0.5105	0.6468	0.0341	2.0608	0.2177	7.1678	0.1354	2.5558
0.3015	0.3281	0.836	0.0103	1.0066	0.0679	11.7235	0.0602	2.3083
0.0057	0.0053	0.0162	0	0.0449	0	3.1199	0.0217	1.2973
0.315	0.3475	5.0302	0	2.1001	0.0217	3.4863	0.0213	2.7012
0	0	0	0	0.0177	0	0	0	0.4763
0.1705	0.3436	1.4793	0.0194	1.573	0.1125	4.0153	0.0714	1.4051
0	0	0	0	0.0246	0	4.3567	0.0269	2.288
0.2	0.4517	0.6429	0.0292	1.6341	0.1631	4.3225	0.0918	1.7359

0.2544	0.566	0.6302	0.0373	2.0918	0.2078	6.5616	0.1377	2.4967
0	0	0.0172	0	0.043	0	1.9733	0.0071	0.4225
0.0078	0.0161	0.0502	0	0.3438	0.0247	11.3318	0.0477	3.1488
0.3377	0.772	0.9421	0.0535	2.8488	0.3079	9.0423	0.1811	3.6461
0.4189	0.7457	1.6791	0.0571	3.2559	0.307	8.9815	0.1683	3.1303
0.3124	0.4868	1.3231	0.0323	2.0383	0.187	6.034	0.107	2.1216
0.3909	0.8807	1.1828	0.0599	3.6114	0.3667	10.493	0.2557	3.8643
0.4922	0.5774	2.1978	0.0247	2.0062	0.131	6.0955	0.0887	2.316
0.1084	0.2293	0.4049	0.0148	0.9137	0.0866	3.3998	0.0742	1.4522
0.2368	0.3161	3.1687	0.0121	1.7176	0.056	3.2843	0.0358	1.5768
0.1005	0.2194	0.3641	0.0142	0.9426	0.0945	3.1135	0.0487	0.9094
0	0.0234	0.024	0	0.3541	0.0103	7.2094	0.062	4.8247
0	0	0.0079	0	0.0409	0	3.3628	0.0183	1.0821
0.1449	0.123	0.6498	0	0.3886	0.0157	0	0.0103	0.3198
0.1153	0.1078	0.1594	0	0.239	0.0207	2.1472	0.018	0.7258
0.0262	0.0302	0.1101	0	0.1008	0.0091	1.0993	0.0102	0.4333

0.0218	0.0245	0.0897	0	0.0789	0	0.8573	0.0071	0.3392
0.0701	0.061	0.3128	0	0.1927	0.0099	1.9109	0.0117	0.5841
0.316	0.35	1.4949	0.008	1.2169	0.0753	3.8042	0.051	2.0681
0.1255	0.2678	0.3394	0.019	1.0194	0.1093	5.0132	0.0713	2.5341
0.1347	0.2942	0.3641	0.0206	1.1525	0.1218	5.6397	0.0817	2.7258
0.0482	0.098	0.1805	0	0.4064	0.0397	3.4088	0.033	1.1496
0.6005	0.5291	3.8364	0.0105	2.0548	0.0582	9.2758	0.0548	2.3893
0.2723	0.2123	1.4777	0	0.7671	0.0188	0	0.028	1.5141
0.1419	0.1493	1.1743	0.0043	0.8064	0.0354	7.8531	0.0505	1.736
0	0	0	0	0.0101	0	0	0.0045	0.1723
0	0	0.0029	0	0.0502	0	1.0041	0.0078	0.5005
0.0485	0.094	0.1705	0.0043	0.4032	0.0367	3.4088	0.032	1.1396

SFA	SFA	SFA	SFA	SFA	**MUFA**	**MUFA**	**MUFA**	**MUFA**
Methyl arachidate（C_{20}:0）	Methyl heneicosanoate（C_{21}:0）	Methyl heneicosanoate（C_{21}:0）	Methyl tricosanoate（C_{23}:0）	Methyl lignocerate（C_{24}:0）	Methyl (Z) - 9 - tetradecenoate（C_{14}:1）	Methyl (Z) - 10 - pentadecenoate（C_{15}:1）	Methyl (Z) - 9 - hexadecenoate（C_{16}:1）	Methyl (Z) - 10 - heptadecenoate（C_{17}:1）
0.3178	0	0	0.0237	0.0649	0.015	0	0.0758	0.013
0.046	0.014	0.014	0.005	0	0.0072	0	0.0682	0.0072
0.1768	0.0109	0.0109	0.015	0.0256	0.0072	0	0.0565	0.0116
0.1086	0.0242	0.0242	0	0	0.0173	0	1.1127	0.0132
0.0493	0.1619	0.1619	0	0	0.1284	0	0.398	0.0462
0.2304	0.3989	0.3989	0	0	0.0558	0	0.2105	0.0255
0.2092	0.1768	0.1768	0	0	0.0596	0	0.1818	0.0095
0.13	0	0	0	0	0.0187	0	0.086	0
0.0297	0.0227	0.0227	0.0155	0	0.0134	0	0.139	0.0143
0.1053	0.1382	0.1382	0	0	0	0	0.052	0.0059
0.0317	0.2408	0.2408	0.0749	0	0.1712	0	0.569	0.0565
0.2075	0.0743	0.0743	0	0	0.0535	0	0.181	0.0255
0.3285	0	0	0.0571	0	0.0179	0	0.1446	0.0169
0.1316	0	0	0.0275	0	0.0207	0	0.0992	0.0065
0	0	0	0.0566	0	0.0245	1.344	0.1222	0
0.0231	0.0754	0.0754	0	0	0.0833	0	0.2376	0.0235
0.5198	0	0	0	0	0.0311	0	0.0405	0.0139
0.0196	0.1856	0.1856	0	0	0.1269	0	0.2793	0.0389

0.0367	0.1063	0.1063	0.0261	0	0.181	0	0.4217	0.0529
0.0577	0	0	0	0	0	0	0.0077	0
0.1046	0	0	0	0.0188	0	0	0.261	0.025
0.0592	0.2821	0.2821	0.0201	0	0.2347	0	0.5345	0.078
0.0456	0.4764	0.4764	0.035	0	0.3179	0	0.7209	0.0838
0.0501	0.2846	0.2846	0.0273	0	0.1961	0	0.4673	0.0539
0.0678	0.2644	0.2644	0.03	0	0.3013	0	0.8132	0.0995
0.0799	0.0696	0.0696	0.058	0	0.1274	0	0.3888	0.0436
0.025	0.041	0.041	0.0066	0	0.0853	0	0.23	0.0312
0.0239	0.065	0.065	0.0185	0	0.0485	0	0.1506	0.0145
0	0.1256	0.1256	0.0072	0	0.0794	0	0.1855	0.0141
0.0771	0	0	0.0338	0	0.029	0	0.5242	0.0403
0.1429	0	0	0.0243	0	0.0291	0	0.0585	0.0081
0.0119	0	0	0	0	0.0207	0.872	0.0404	0
0.0699	0	0	0.1071	0	0.027	0	0.4844	0
0.0425	0	0	0	0.0353	0.018	0	0.0336	0.0044

0.0293	0	0	0	0	0.0265	0	0.0194	0
0.0544	0	0	0	0	0.0217	0	0.0363	0.0055
0.0434	0.0242	0.0242	0.0169	0	0.0653	0	0.1822	0.0216
0.0638	0.069	0.069	0.028	0	0.1071	0	0.2576	0.0276
0.2215	0.0772	0.0772	0.0276	0.0322	0.1074	0	0.9566	0.0347
0.0289	0.0471	0.0471	0.0627	0	0.0297	0	0.2154	0.0113
0.0696	0.0463	0.0463	0.0425	0	0.0365	0	0.2541	0.0164
0.0989	0.012	0.012	0.0091	0	0.0062	7.0327	0.0747	0.0089
0.0912	0.0292	0.0292	0	0	0.0149	0	0.1019	0.0148
0.033	0	0	0	0	0	0.356	0.0115	0.0061
0.0188	0	0	0	0	0	0	0.0866	0.0054
0.0289	0.0461	0.0481	0.0627	0	0.0297	0	0.2154	0.0113

MUFA	MUFA	MUFA	MUFA	MUFA	n-6	n-6	n-6	n-6
Methyl (E) - 9 - octadecenoate (C_{18}: 1n9t)	Methyl (Z) - 9 - octadecenoate (C_{18}: 1n9c)	Methyl (Z) - 11 - eicosenoate (C_{20}: 1)	Methyl (Z) - 13 - docosenoate (C_{22}: 1n9)	Methyl (Z) - 15 - tetracosenoate (C_{24}: 1)	Methyl (E, E) - 9, 12 - octadecadienoate (C_{18}: 2n6t)	Methyl (Z, Z) - 9, 12 - octadecadienoate (C_{18}: 2n6c)	Methyl (Z, Z, Z) - 6, 9, 12 - octadecatrienoate (C_{18}: 3n6)	Methyl (Z, Z, Z) - 8, 11, 14 - eicosatrienoate (C_{20}: 3n6)
0.0283	10.2551	0.1869	0	0	0.0003	10.8511	0.0648	0.0322
0.0461	5.2959	0.0363	0	0	0	2.2573	0.0111	0.0064
0.0302	5.09	0.0704	0	0	0	8.0287	0.0317	0.0348
0.1008	5.9403	0.0763	0	0	0	6.7778	0.0393	0
0.3756	7.1237	0.0409	0.027	0	0	2.2758	0.0136	0
0.2105	6.8235	0.0197	0	0	0	3.1977	0	0
0.072	18.1367	0.0166	0.0145	0	0	4.7644	0	0.0147
0.0642	4.1548	0.0269	0	0	0	1.6817	0	0
0.064	3.7622	0.0438	0.0176	0	0	2.3708	0.009	0.0108
0.5352	9.4442	0.0101	0	0	0	2.825	0	0.0128
0.5057	0.7546	0.0418	0	0	0.0339	2.5422	0.0187	0
0.1907	13.3705	0.0877	0	0	0.1885	7.0163	0.0356	0.0776
0.0194	7.7988	0.107	0	0	0	13.1273	0.0566	0.0634
0.0566	4.1447	0.0351	0	0	0	3.6942	0.0191	0.0142
0	2.1534	0.0176	0	0	0	1.7791	0	0
0.221	2.8681	0	0	0	0	0.487	0	0
0.0371	15.3507	0.1461	0	0.244	0.1	21.193	0.0579	0.075
0.505	3.0948	0	0	0	0	1.0285	0	0

0.3468	5.5132	0.0365	0.0166	0	0.081	1.9022	0	0.0113
0	2.685	0.0262	0	0	0	2.5099	0	0.0234
0.047	15.2996	0.1362	0	0	0	4.3397	0.0123	0.0089
0.7276	7.0273	0.0154	0	0	0.1713	0.7417	0	0
0.8477	7.3551	0.0547	0	0	0.0956	2.5506	0.0174	0.0258
0.5469	5.2842	0.0453	0	0	0.0594	2.2656	0	0.0179
0.9416	8.5663	0.0696	0	0	0.1591	2.3938	0.0165	0.0202
0.1937	6.7249	0.0746	0.0113	0	0.0954	4.7688	0.0294	0.0275
0.182	3.1481	0.0286	0	0	0.0493	1.3789	0	0
0.1563	3.072	0.0097	0	0	0.0523	1.388	0	0.0079
0.2419	2.0777	0	0	0	0.0105	0.733	0	0.0093
0.0634	13.692	0.2511	0.0149	0	0.0419	3.9602	0	0
0.0185	6.2544	0.1061	0	0	0.0929	9.3521	0.0543	0.0381
0.0328	1.1292	0.021	0	0	0	1.244	0.0105	0
0.0487	5.3698	0.0853	0.0139	0	0	2.0258	0	0.0212
0.0195	2.766	0.0494	0	0	0.0467	3.8087	0.0283	0.0147

0.0146	1.5751	0.0323	0	0	0.035	3.2039	0.0218	0.0094
0.0218	3.0157	0.0392	0	0	0.0339	2.3785	0.0201	0.0086
0.1665	3.8576	0.0273	0	0	0.0542	2.0881	0	0.0101
0.2712	5.016	0.0373	0	0	0.0668	2.7069	0	0.0137
0.2941	7.9008	0.1242	0.0092	0	0.0711	2.5684	0	0.0364
0.084	4.6831	0.0382	0	0	0	4.3592	0.0186	0.0194
0.1461	8.4116	0.0594	0.0061	0	0	3.4506	0.0186	0.0147
0.0495	7.8502	0.0702	0	0	0	4.9158	0.0229	0.0196
0.145	7.5366	0.0454	0	0	0	3.1017	0.0159	0.0096
0.0068	2.6654	0.0603	0	0	0.0095	1.4358	0.0074	0.0087
0.0111	2.607	0.0576	0	0	0.0112	0.955	0	0
0.0842	4.6821	0.0382	0	0	0	4.3592	0.0186	0.0184

n-6	n-6	n-6	n-6	n-3	n-3	n-3	n-3	n-3
Methyl (Z，Z，Z，Z) －5，8，11，14 －eicosatetraenoate（C_{20}:4n6）	Methyl (Z，Z) －13，16 －docosadienoate（C_{22}:2）	Methyl tetracosatetraenoate（C_{22}:4）	Methyl (Z，Z，Z，Z，Z) －4，7，10，13，16 －docosapentaenoate（C_{22}:5n6）	Methyl (Z，Z，Z) －9，12，15 －octadecatrienoate（C_{18}:3n3）	Methyl (Z，Z，Z，Z，Z) －5，8，11，14，17 －eicosapentaenoate（C_{20}:5n3）	Methyl (Z，Z，Z，Z，Z) －5，8，11，14，17 －eicosapentaenoate（C_{20}:5n3）	Methyl (Z，Z，Z，Z，Z，Z) －4，7，10，13，16，19 －docosahexaenoate（C_{22}:6n3）	Methyl (Z，Z，Z，Z，Z) －7，10，13，16，19 －docosapentaenoate（C_{22}:5n3）
0	0	0	0	0.54	0	0	0	0
0	0	0	0	0.1088	0	0	0	0
0	0	0	0	0.569	0	0	0	0
0	0	0	0	0.6314	0	0	0	0
0	0.0962	0	0.0219	0.1738	0	0	0	0
0.02964	0	0	0	0.0357	0	0	0	0
0	0.0542	0.0108	0	0.0582	0	0	0	0
0	0	0	0	0.0761	0	0	0	0
0	0	0	0	0.2169	0	0	0	0
0	0	0	0	0.0302	0	0	0	0
0	0	0	0	0.2644	0	0	0	0
0	0	0	0	0.4827	0	0	0	0
0	0	0	0	1.2271	0	0	0	0
0	0	0	0	0.3039	0	0	0	0
0	0	0	0	0.1146	0	0	0	0
0	0	0	0	0.0514	0	0	0	0
0	0	0	0	1.311	0.0656	0.0656	0	0
0	0	0	0	0.1657	0	0	0	0

0	0	0	0	0.0836	0	0	0	0
0	0	0	0	0.6441	0	0	0	0
0	0	0	0	0.1058	0	0	0	0
0	0	0	0	0.1375	0	0	0	0
0	0.0134	0	0	0.3404	0	0	0	0
0	0	0	0	0.2699	0	0	0	0
0	0.0137	0	0	0.3412	0	0	0	0
0	0	0	0.0274	0.4221	0	0	0	0.0167
0	0	0	0	0.1452	0	0	0	0
0	0	0	0	0.1558	0	0	0	0
0	0	0	0	0.0877	0	0	0	0
0	0	0	0	0.1358	0	0	0.0174	0
0	0	0	0	0.8413	0.0229	0.0229	0	0
0	0	0	0	0.1664	0.0141	0.0141	0	0
0	0	0	0	0.315	0.0391	0.0391	0	0
0	0	0	0	0.4441	0	0	0	0

0	0	0	0	0.3874	0	0	0	0
0	0	0	0	0.2534	0	0	0	0
0	0	0	0	0.1755	0	0	0	0
0	0	0	0	0.2429	0	0	0	0
0	0	0	0	0.2066	0	0	0	0
0	0	0	0.0366	0.296	0.017	0.017	0	0
0	0	0	0.0424	0.1848	0	0	0	0.0119
0	0	0	0	0.312	0	0	0	0
0	0	0	0	0.1508	0	0	0	0
0.0278	0	0	0	0.3761	0	0	0	0
0	0	0	0	0.1204	0	0	0	0
0	0	0	0.0366	0.296	0.017	0.017	0	0

Total Fatty Acids (g/100g)	SFA(g/100g)	TFA(g/100g)	MUFA/(g/100g)	PUFA/(g/100g)	n-6PUFA	n-3PUFA	AI	TI
27.2866	5.2241	0.0286	10.5741	11.4884	10.9484	0.54	0.19	0.39
15.6912	7.8467	0.0461	5.4609	2.3836	2.2748	0.1088	0.42	1.62
17.9762	4.0461	0.0302	5.2659	8.6642	8.0952	0.569	0.11	0.42
17.4854	2.7763	0.1008	7.2606	7.4485	6.8171	0.6314	0.24	0.21
27.5883	16.8672	0.3756	8.1398	2.5813	2.4075	0.1738	1.45	2.01
29.74844	19.1399	0.2105	7.3455	3.26304	3.22734	0.0357	1.65	2.30
41.2443	17.8513	0.072	18.4907	4.9023	4.8441	0.0582	0.14	1.43
12.6386	6.5302	0.0642	4.3506	1.7578	1.6817	0.0761	0.49	1.69
11.3702	4.7084	0.064	4.0543	2.6075	2.3906	0.2169	0.36	1.08
23.8207	10.9053	0.5352	10.0474	2.868	2.8378	0.0302	0.22	1.50
20.2911	15.3331	0.5396	2.0988	2.8592	2.5948	0.2644	2.31	3.64
39.106	17.3964	0.3792	13.9089	7.8007	7.318	0.4827	0.33	1.24
27.4947	4.9157	0.0194	8.1046	14.4744	13.2473	1.2271	0.07	0.31
22.6939	14.2997	0.0566	4.3628	4.0314	3.7275	0.3039	1.92	1.64
6.106	0.5506	0	3.6617	1.8937	1.7791	0.1146	0.10	0.16
14.1538	10.1819	0.221	3.4335	0.5384	0.487	0.0514	2.31	3.15
45.9475	7.216	0.1371	15.8634	22.8681	21.4259	1.4422	0.06	0.29
16.2417	11.0026	0.505	4.0449	1.1942	1.0285	0.1657	1.70	2.41

23.6715	15.0247	0.4278	6.5687	2.0781	1.9945	0.0836	1.33	2.44
8.4171	2.5208	0	2.7189	3.1774	2.5333	0.6441	0.10	0.51
35.3298	15.0943	0.047	15.7688	4.4667	4.3609	0.1058	0.23	1.42
30.6442	20.9762	0.8989	8.6175	1.0505	0.913	0.1375	1.65	2.92
34.6787	22.2554	0.9433	9.3801	3.0432	2.7028	0.3404	1.44	2.14
23.8098	14.6033	0.6063	6.5937	2.6128	2.3429	0.2699	1.26	1.89
38.0716	24.3356	1.1007	10.7915	2.9445	2.6033	0.3412	1.42	2.29
28.1201	15.1685	0.2891	7.5643	5.3873	4.9485	0.4388	0.97	1.36
12.7266	7.448	0.2313	3.7052	1.5734	1.4282	0.1452	1.04	1.86
16.0871	11.0315	0.2086	3.4516	1.604	1.4482	0.1558	2.30	2.17
9.9918	6.5527	0.2524	2.5986	0.8405	0.7528	0.0877	1.47	2.42
31.389	12.6188	0.1053	14.6149	4.1553	4.0021	0.1532	0.33	1.26
21.5784	4.6792	0.1114	6.4747	10.4245	9.5374	0.8871	0.07	0.42
5.3423	1.7771	0.0328	2.1161	1.4491	1.2545	0.1946	0.71	0.29
12.3028	3.8335	0.0487	6.0291	2.4402	2.047	0.3932	0.22	0.58
9.1885	1.9551	0.0662	2.8909	4.3425	3.8984	0.4441	0.13	0.34

6.8232	1.4978	0.0496	1.6679	3.6575	3.2701	0.3874	0.14	0.34
9.0996	3.2649	0.0557	3.1402	2.6945	2.4411	0.2534	0.29	0.74
16.7301	10.0817	0.2207	4.3205	2.3279	2.1524	0.1755	1.27	1.84
19.2115	10.4644	0.338	5.7168	3.0303	2.7874	0.2429	0.79	1.69
24.2159	11.9064	0.3652	9.427	2.8825	2.6759	0.2066	0.63	1.41
15.4986	5.6731	0.084	5.0617	4.7638	4.4338	0.33	0.30	0.85
32.0066	19.3534	0.1461	8.9302	3.723	3.5263	0.1967	1.14	2.00
24.8469	4.4842	0.0495	15.0924	5.2703	4.9583	0.312	0.30	0.21
23.3371	12.2005	0.145	7.8586	3.278	3.1272	0.1508	0.55	1.73
5.1913	0.2199	0.0163	3.1061	1.8653	1.4892	0.3761	0.04	0.05
5.4386	1.5843	0.0223	2.7677	1.0866	0.9662	0.1204	0.18	0.66
15.4803	5.6566	0.0842	5.0609	5.0312	9.3904	9.1936	9.2007	9.1165

Glucose	Fructose	ose (g/1	Maltose	Lactose(	The total sugar (g/100g)
0.181	0.278403	0.570	1.873	—	2.903
1.503643	1.253	2.059	0.695369	0.357305	5.869
0.848	0.642	2.327	0.389	—	4.206
0.199173	0.281	6.863	1.122	0.193237	8.659
0.360527	—	—	—	0.235	0.595
0.252	—	—	—	0.213543	0.465
—	0.220	0.225888	—	—	0.445
1.24503	0.989203	3.388	0.519343	0.443386	6.585
—	0.260	2.311	0.54509	—	3.116
—	—	8.439562	0.183	0.103	8.725
0.248787	—	0.207	—	—	0.456
—	—	1.964	—	—	1.964
—	2.325	—	0.416517	—	2.742
—	—	0.572	—	—	0.572
0.443	0.564721	26.39447	—	—	27.402
1.124142	—	—	5.923	0.510549	7.557
—	0.19998	1.476255	—	—	1.676235
0.444	0.514	0.637505	—	—	1.596

—	0.379831	0.293	0.311892	—	0.985
0.648	—	—	0.291342	—	0.939
—	—	3.298	0.110	—	3.408
—	0.236208	0.588174	0.211	—	1.035
—	—	—	—	—	—
0.263	0.146524	—	—	—	0.409
—	—	0.290	—	—	0.290
0.288	—	—	—	—	0.288
1.051587	—	—	0.678463	0.272116	2.002
0.286026	—	—	—	0.136245	0.422
2.187	1.357	0.944	1.624	1.341	7.452
0.184	—	2.816	—	—	3.000
—	—	0.586	—	—	0.586
0.160	—	0.895	1.501	—	2.557
—	—	0.552	—	—	0.552
—	—	0.824	—	—	0.824

0.213	—	1.223735	—	—	1.437
0.403	—	3.794384	—	—	4.197
0.24503	—	1.079591	0.504571	—	1.829192
—	—	0.641228	0.452715	—	1.093942
—	—	0.754233	0.469014	—	1.223247
0.900	0.311	—	0.381	0.433	2.025
0.793	0.606	1.857	0.553	—	3.809
—	—	7.298496	0.456086	—	7.755
0.603	0.512893	1.281421	0.838807	1.147	4.383
0.296148	0.916647	—	—	—	1.212794
0.2252	—	—	—	—	0.2252
0.890	0.331	—	0.371	0.463	2.055
